# Activity-function transitions and interpretable machine learning for predicting incident depressive symptoms among ACE-exposed middle-aged and older adults: a multi-cohort study

**DOI:** 10.3389/fpubh.2026.1924836

**Published:** 2026-09-03

**Authors:** Zhenhao Lin, Yuwen Shangguan, Young-Je Sim, Dahua Chen, Xiang Wang

**Affiliations:** 1 Department of Pediatrics, Binhai County People’s Hospital, Yancheng, Jiangsu, China; 2 Department of Exercise Physiology, Kunsan National University, Gunsan, Republic of Korea; 3 School of Rehabilitation Medicine, Jining Medical University, Jining, Shandong, China

**Keywords:** adverse childhood experiences, depression, longitudinal cohort, machine learning, middle-aged and older adults, physical activity

## Abstract

**Background:**

Ad--verse childhood experiences (ACEs) are associated with increased risk of depressive symptoms in later life. This study aimed to develop and externally validate an interpretable machine-learning model for predicting incident depressive symptoms among ACE-exposed middle-aged and older adults.

**Methods:**

Data were obtained from the English Longitudinal Study of Aging (ELSA) and the Health and Retirement Study (HRS). Participants with baseline depressive symptoms were excluded. ELSA was used for model development and HRS for external validation. LASSO regression was applied for predictor selection, followed by comparison of multiple machine-learning algorithms. Pre-outcome activity-function transition profiles were further examined in relation to observed incident depressive symptoms.

**Results:**

LASSO identified stable predictors mainly involving physical function, physical activity, chronic conditions, and sociodemographic characteristics. Among the evaluated models, random forest demonstrated the best performance, with an AUC of 0.701 in ELSA and 0.693 in HRS external validation. SHAP analysis identified walking time, grip strength, falls, arthritis, and physical activity as important contributors to prediction. Participants with unfavorable activity-function status at both pre-outcome assessments showed higher odds of incident depressive symptoms in both cohorts.

**Conclusion:**

An interpretable machine-learning framework integrating physical performance, physical activity, and health-related factors achieved moderate and externally validated prediction of incident depressive symptoms among ACE-exposed adults. Pre-outcome activity-function transition profiles provided additional information on subsequent depressive-symptom risk. Given the moderate discrimination and limited case-detection ability, the framework should be regarded as exploratory and requires further validation and refinement before any clinical application.

## 1 Introduction

Depressive symptoms are a common and important mental health problem among middle-aged and older adults and are closely associated with reduced quality of life, increased chronic disease burden, physical functional limitations, greater use of health care services, and adverse health outcomes (1, 2). As global population aging accelerates, early identification of individuals at high risk of depression during midlife and later life has become an important issue in clinical medicine and public health (3, 4). In particular, among populations with long-term psychosocial vulnerability, the development of interpretable and generalizable risk prediction tools is of considerable importance for improving screening efficiency and informing individualized intervention strategies (5).

Adverse childhood experiences (ACEs) are recognized as important early-life risk factors that influence health across the life course (6). Early adverse experiences, including abuse, neglect, household dysfunction, parental separation, and economic hardship, have been associated with depressive symptoms, chronic diseases, and functional decline in adulthood and later life (7, 8). For individuals exposed to childhood adversity, mental health risks in midlife and later life may be shaped not only by early-life experiences but also by health behaviors, social support, chronic disease status, and changes in physical function across the subsequent life course (9, 10). Therefore, focusing on middle-aged and older adults with a history of ACEs may help identify clinically meaningful and measurable predictors within this potentially high-risk population.

Physical activity and physical function are important components of health assessment among middle-aged and older adults. Previous studies have suggested that higher levels of physical activity are generally associated with a lower risk of depressive symptoms (11, 12). Meanwhile, indicators of physical function, such as walking time, grip strength, balance ability, limitations in activities of daily living/instrumental activities of daily living (ADL/IADL), and falls, may also reflect an individual’s health reserves, capacity for independent living, and level of social participation (13). However, most existing studies have focused on physical activity or physical function assessed at a single time point, and limited evidence is available regarding the longitudinal patterns of these factors over extended follow-up periods and their relationship with depression risk prediction. In particular, among middle-aged and older adults with a history of ACEs, whether changes in physical activity and physical performance across repeated pre-outcome assessments can provide additional information for depression risk characterization remains to be further investigated.

Recent studies have increasingly applied machine-learning and deep-learning approaches to depression prediction in aging populations. Wang et al. (14) applied deep-learning algorithms to a large CHARLS sample to examine depression prediction at both population and individual levels. Liu et al. (5) subsequently developed a transcultural prediction framework using HRS and CHARLS, combining feature selection, multiple machine-learning algorithms, independent cross-cohort validation, calibration assessment, decision curve analysis, and SHAP-based interpretation. More recently, Long et al. (15) leveraged five waves of CHARLS data to develop a dual-attention residual network following LASSO-based predictor screening and independently validated the model in CLHLS. Together, these studies demonstrate that machine-learning prediction, external validation, and explainable modelling are increasingly established components of late-life depression research rather than methodological innovations in themselves. However, the present study addresses a more specific question that remains less well characterized: prediction of incident depressive symptoms among middle-aged and older adults with a history of adverse childhood experiences. Individuals with ACE exposure represent a psychosocially vulnerable but heterogeneous population in whom later-life depressive symptoms may reflect the combined influence of health behaviors, chronic conditions, sociodemographic circumstances, and physical performance. In addition, prediction models may be difficult to interpret when they rely heavily on subjective health, social, or functional measures that are conceptually close to depressive symptoms themselves. We therefore sought to examine whether a more parsimonious set of physical-performance, physical-activity, chronic-disease, behavioral, and sociodemographic predictors could support reproducible prediction across independent aging cohorts. Given the limited number of repeated predictor assessments available for the primary prediction analysis, we prioritized parsimonious and interpretable feature selection rather than a complex sequence-based modelling architecture. Based on this rationale, the present study used two large longitudinal cohorts, the English Longitudinal Study of aging (ELSA) and the Health and Retirement Study (HRS), to develop and validate a depression risk prediction framework among middle-aged and older adults with a history of ACEs, based on approximately 8 years of follow-up data. Specifically, LASSO penalized logistic regression was used to identify a parsimonious set of predictors in the ELSA development cohort. Second, multiple machine-learning models were compared using internal validation in ELSA, followed by independent external validation in HRS. Third, SHAP-based interpretability analysis was applied to identify the main variables contributing to depression risk prediction. Fourth, pre-outcome activity-function transition profiles defined across two assessments were examined in relation to subsequently observed incident depressive symptoms. Through these analyses, we aimed to evaluate the cross-cohort generalizability and interpretability of depression-risk prediction in ACE-exposed middle-aged and older adults and to characterize whether pre-outcome changes in activity-function status were associated with subsequent depressive symptoms.

## 2 Materials and methods

### 2.1 Study design and population

**Figure 1:**
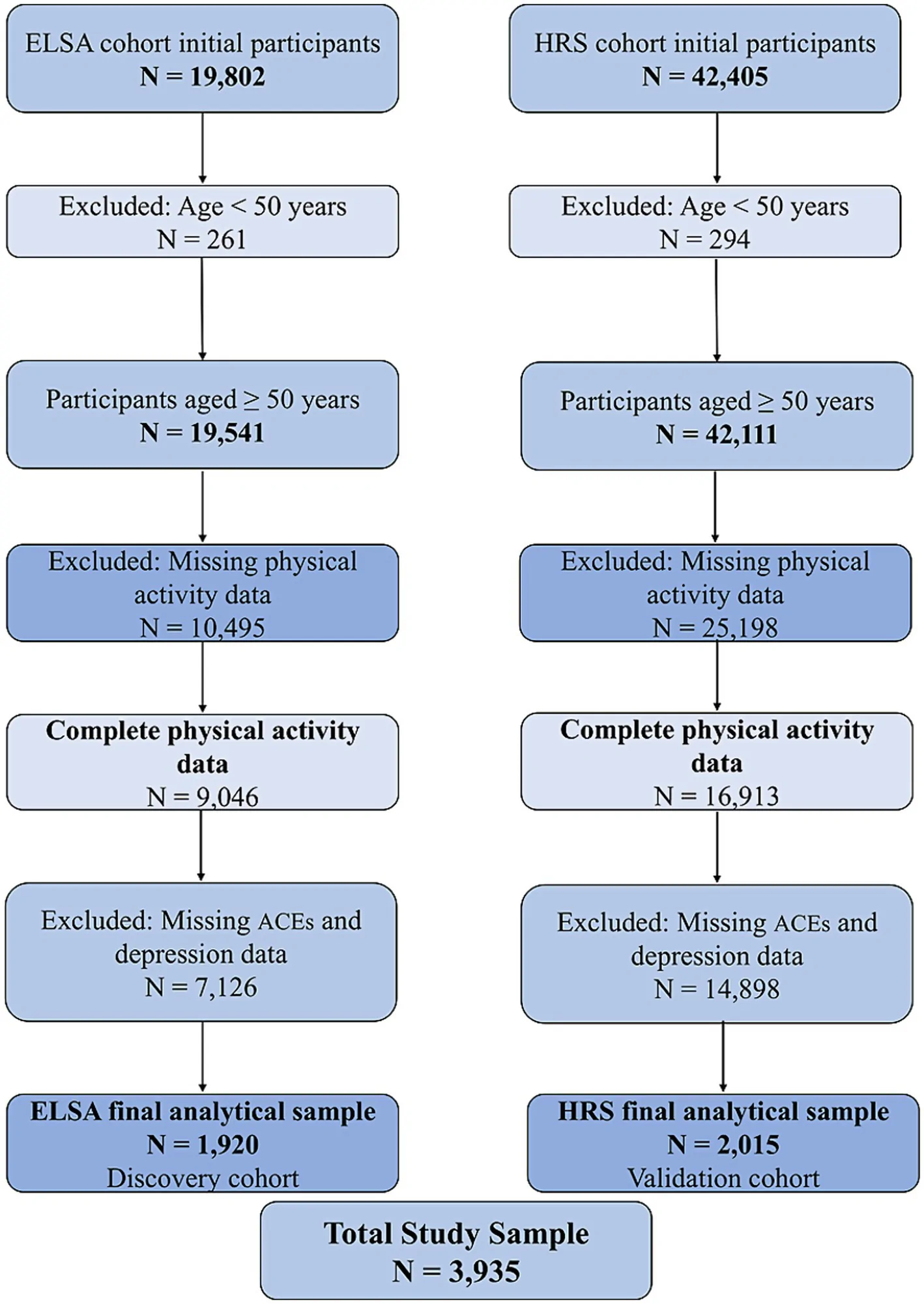
Flow diagram for selecting ACE-exposed participants from the ELSA and HRS cohorts.

This study used a longitudinal prognostic modelling design based on two aging cohorts. The English Longitudinal Study of aging (ELSA) was used for model development, and the Health and Retirement Study (HRS) was used for independent external validation (16, 17). In ELSA, Wave 2 was treated as baseline, Wave 4 as the pre-outcome follow-up wave, and Wave 6 as the outcome wave. In HRS, the corresponding waves were Wave 9, Wave 11, and Wave 13. For the primary prediction analysis, candidate predictors were obtained from baseline (Wave 2 in ELSA and Wave 9 in HRS), whereas depressive symptoms were assessed at the final outcome wave (Wave 6 in ELSA and Wave 13 in HRS). The intermediate pre-outcome assessments (Wave 4 in ELSA and Wave 11 in HRS) were used separately for the activity-function transition analysis described in Section 2.7. The general cross-cohort harmonization, missing-data strategy, and external validation framework were adapted from our previous multi-cohort modelling study (18). However, the present analysis differed from that work by focusing specifically on participants with reported adverse childhood experiences and by examining incident depressive symptoms rather than depression-cognition outcomes. Participants were excluded if they were younger than 50 years, had depressive symptoms at baseline, had no reported ACE exposure, or had missing information on physical activity, ACEs, or depressive symptom assessment. The final analytic sample included 1,920 participants from ELSA and 2,015 participants from HRS. The participant selection process is shown in Figure 1. Variables with more than 20% missing data were removed before modelling. Missing values in the remaining predictors were imputed using multiple imputation by chained equations with predictive mean matching, generating 20 imputed datasets. To characterize potential selection associated with predictor missingness, baseline characteristics were additionally compared between participants with complete predictor data and those with at least one predictor value requiring imputation. Differences were summarized using absolute standardized mean differences (SMDs). These comparisons are presented separately for ELSA and HRS in Supplementary Tables 13 and 14. To reduce information leakage, imputation models in internal validation were fitted within the training folds and then applied to the corresponding validation folds. For external validation, the HRS data were imputed separately and were not used during ELSA model training, feature selection, or hyperparameter tuning.

### 2.2 Outcome definition and ACE exposure

The outcome was incident depressive symptoms at the final follow-up assessment. Depressive symptoms were measured using the eight-item Center for Epidemiologic Studies Depression Scale (CES-D-8) in both cohorts. Consistent with previous aging cohort studies, a CES-D-8 score of 3 or higher was used to indicate depressive symptoms, whereas scores below 3 were classified as no depressive symptoms (19). Because participants with depressive symptoms at baseline had been excluded, the outcome in this study reflected newly identified depressive symptoms during follow-up. ACE exposure was used to define the target analytic population. Participants were included if they reported at least one adverse childhood experience. Because ELSA and HRS used different childhood adversity questionnaires, ACE exposure was harmonized as the presence of one or more cohort-specific ACE indicators. Both cohorts included 12 ACE-related indicators, covering domains such as childhood abuse, parental separation or loss, household dysfunction, institutional or foster care experience, childhood poverty or neglect, and adverse family relationships (20–22). The full cohort-specific ACE definitions and item mappings are provided in Supplementary Table 1. The binary ACE definition was used primarily to define a harmonized ACE-exposed target population across cohorts and was not intended to imply equivalence across adversity subtypes or levels of cumulative exposure. To examine cumulative burden, ACE exposure was additionally categorized as 1, 2, or ≥3 reported ACEs in secondary analyses. Associations between these ACE-burden categories and incident depressive symptoms were examined separately in ELSA and HRS, together with an ordinal trend across categories. Because the specific ACE items and questionnaire structures were not identical between cohorts, these burden analyses were interpreted as cohort-specific exploratory analyses rather than directly interchangeable quantitative dose measures.

### 2.3 Candidate predictor variables

Candidate predictors were selected according to previously reported factors associated with depressive symptoms in middle-aged and older adults (23, 24). The candidate variables covered sociodemographic characteristics, socioeconomic status, health behaviors, chronic conditions, physical performance, and physical activity. To reduce conceptual overlap with the CES-D-8 outcome, self-rated health, social activity, and ADL/IADL limitations were excluded *a priori* from the candidate predictor set before feature selection. To preserve temporal ordering and avoid using information measured concurrently with the outcome, only variables collected before the final outcome assessment were considered. For cross-cohort analysis, ELSA and HRS variables were recoded into comparable analytic categories when their meanings were sufficiently similar. Education and employment were grouped into broad categories that could be aligned across cohorts. Physical activity indicators were derived from cohort-specific frequency questions and coded as participation at least weekly versus less than weekly, following previous ELSA-based physical activity studies (25, 26). Detailed variable definitions and cohort-specific coding rules are provided in Supplementary Table 1.

### 2.4 LASSO-based feature selection

Participants with baseline depressive symptoms (CES-D-8 ≥ 3) were excluded before feature selection to ensure that the outcome represented incident depressive symptoms. ELSA served as the development cohort, with Wave 2 predictors used to predict Wave 6 outcomes, whereas HRS was reserved for external validation using Wave 9 predictors and Wave 13 outcomes. Using the 20 multiply imputed datasets described in Section 2.1, LASSO penalized logistic regression was fitted separately in each dataset. During internal validation, imputation, dummy-variable encoding, standardization, and feature selection were repeated within each outer training fold without access to the corresponding validation fold. The penalty parameter was selected using stratified 10-fold inner cross-validation and the one-standard-error rule. Predictors with non-zero coefficients in at least 10 of the 20 imputed datasets were retained. A categorical predictor was considered selected when at least one of its dummy-variable coefficients was non-zero. Subsequent prediction models were fitted separately across the 20 imputed datasets, and participant-level predicted probabilities were averaged across models rather than stacking the datasets. Stratification preserved the outcome proportion within each fold, and no over- or undersampling was applied.

### 2.5 Machine learning classification

The 11 selected predictors were entered into 12 candidate classifiers for binary prediction of incident depressive symptoms. The candidate models included logistic regression, linear discriminant analysis, quadratic discriminant analysis, random forest, gradient boosting, XGBoost, LightGBM, AdaBoost, support vector machine, naive Bayes, decision tree, and k-nearest neighbors. For all prediction models, predictor inputs were restricted to baseline measurements. The intermediate pre-outcome assessments were not incorporated into the machine-learning feature vector and were used separately only for the activity-function transition analysis described in Section 2.7. The final outcome wave was used only to define incident depressive-symptom status. Feature scaling parameters were estimated from the ELSA training data and then applied to the HRS cohort. Internal validation was performed in ELSA using stratified five-fold cross-validation, and each model was subsequently refitted in the full ELSA sample and evaluated in the independent HRS cohort. Model discrimination was assessed using AUC as the primary metric. Accuracy, precision, recall, and F1 score were also calculated to describe classification performance. Confidence intervals for performance metrics were estimated using 1,000 bootstrap resamples. ROC curves, calibration curves, and decision curve analysis were generated for both the ELSA internal validation results and the HRS external validation results.

### 2.6 Interpretability analysis

SHAP analysis was performed for the final selected random forest model to examine how predictors contributed to model-predicted risk of incident depressive symptoms. Global interpretation was based on SHAP summary plots, which were used to rank predictors and describe the direction of their associations with predicted risk. Local explanations were generated using waterfall and force plots to illustrate how individual predictors contributed to the predicted risk for representative participants. SHAP dependence plots were further used to examine whether key continuous variables, such as walking time, showed nonlinear relationships with model-predicted risk. These analyses were descriptive and were intended to improve transparency of the prediction model rather than to infer causal effects (27).

### 2.7 Activity-function transition profile analysis

Among participants without depressive symptoms at baseline, an activity-function composite score was constructed using walking performance and moderate- and vigorous-intensity physical activity measured at two pre-outcome assessments. Walking time was reverse-coded so that higher values represented more favourable performance. Each component was standardized using the cohort-specific baseline mean and standard deviation, and the same baseline parameters were applied to the follow-up measurements. The standardized components were then averaged with equal weights. The cohort-specific baseline median was applied at both assessments to classify participants as having favourable or unfavorable activity-function status. Based on status at the two assessments, participants were classified into four transition profiles: favourable at both assessments, improving (unfavorable to favourable), declining (favourable to unfavorable), and unfavorable at both assessments. These profiles were intended to describe two-point status transitions and were not interpreted as longitudinal trajectories. Profiles were based on Waves 2 and 4 in ELSA and Waves 9 and 11 in HRS, with incident depressive symptoms assessed at Waves 6 and 13, respectively. Observed incidence and Wilson 95% confidence intervals were calculated for each profile. Associations with incident depressive symptoms were evaluated using logistic regression and reported as odds ratios with 95% confidence intervals, adjusting for baseline age, sex, and education. ACE-stratified analyses and likelihood-ratio interaction tests were conducted as exploratory analyses. Analyses including participants with baseline depressive symptoms were performed as sensitivity analyses.

### 2.8 Statistical analysis

All analyses were performed using Python 3.13. Continuous variables were summarized as means with standard deviations or medians with interquartile ranges, and categorical variables as counts and percentages. Group differences were evaluated using t-tests or Mann–Whitney U tests for continuous variables and chi-square or Fisher’s exact tests for categorical variables, as appropriate. Predictive performance was evaluated using out-of-fold predictions in ELSA and independent external-validation predictions in HRS. Metrics included AUC, accuracy, precision, recall, F1 score, and Brier score. Confidence intervals were estimated using 1,000 bootstrap resamples. Calibration curves and decision curve analysis were used to assess calibration and net benefit. For the activity-function transition analysis, observed incidence and Wilson 95% confidence intervals were calculated for each profile. Associations with incident depressive symptoms were examined using unadjusted and adjusted logistic regression and reported as odds ratios with 95% confidence intervals. Adjusted models included baseline age, sex, and education. ACE-stratified analyses were considered exploratory, and interactions between ACE burden and transition profiles were evaluated using likelihood-ratio tests. Analyses including participants with baseline depressive symptoms were conducted as sensitivity analyses. In secondary analyses, cumulative ACE burden was categorized as 1, 2, or ≥3 ACEs. Associations with incident depressive symptoms were estimated using logistic regression with the 1-ACE group as the reference category. An ordinal trend test was additionally performed by treating the three ACE-burden categories as ordered levels. All tests were two-sided, with *p* < 0.05 considered statistically significant.

## 3 Results

### 3.1 Population baseline characteristics

Baseline characteristics were described using data from ELSA Wave 2 and HRS Wave 9, respectively (Supplementary Tables 2 and 5). All participants included in the analytic sample were free of depressive symptoms at baseline. Therefore, baseline characteristics were compared according to incident depressive symptom status at the final follow-up wave. The final analytic sample included 1,920 participants from ELSA, comprising 1,512 participants who remained free of depressive symptoms and 408 who developed incident depressive symptoms by Wave 6. The HRS analytic sample included 2,015 participants, comprising 1,575 participants who remained free of depressive symptoms and 440 who developed incident depressive symptoms by Wave 13. In both cohorts, compared with participants who remained free of depressive symptoms, those who developed incident depressive symptoms had a higher proportion of women, lower educational attainment, and lower frequencies of vigorous- and moderate-intensity physical activity at baseline. They were also more likely to report poor self-rated health, arthritis, hypertension, falls, and limitations in activities of daily living (ADL) and instrumental activities of daily living (IADL). In addition, participants who developed incident depressive symptoms had relatively higher proportions of current smoking, unemployment, and unmarried status. For continuous variables, participants who developed incident depressive symptoms generally showed poorer baseline physical function, including lower grip strength, lower peak expiratory flow, poorer balance ability, and higher BMI. By contrast, cancer did not show significant between-group differences in either cohort. Stroke was not significantly different between groups in ELSA but showed a significant difference in HRS. Overall, baseline findings from both cohorts indicated that subsequent incident depressive symptom status was associated with lower socioeconomic status, lower physical activity levels, poorer health status, and reduced physical function at baseline. Complete baseline characteristics are presented in Supplementary Tables 2–7. Additional analyses indicated that complete predictor data were available for only 277 of 1,920 participants (14.4%) in ELSA and 165 of 2,015 participants (8.2%) in HRS. Participants with complete and incomplete predictor data differed across several baseline characteristics. In ELSA, the largest imbalances were observed for age group, employment status, walking time, and moderate-intensity physical activity, whereas in HRS, prominent differences involved age group, employment status, smoking, sex, walking time, and vigorous-intensity physical activity. The marked age-group imbalance reflected age-dependent administration patterns for selected physical-performance measures. These findings indicated that complete-case restriction would have selected a substantially different subset of participants and supported the use of multiple imputation (Supplementary Tables 13 and 14). In secondary analyses examining cumulative ACE burden, the incidence of depressive symptoms in ELSA increased from 20.4% among participants with 1 ACE to 23.6% among those with 2 ACEs and 26.7% among those with ≥3 ACEs. Compared with the 1-ACE group, the ORs were 1.23 (95% CI: 0.96–1.58; *p* = 0.109) for 2 ACEs and 1.44 (95% CI: 1.00–2.08; *p* = 0.051) for ≥3 ACEs. The ordinal trend across ACE-burden categories was statistically significant (OR per category increase = 1.21, 95% CI: 1.03–1.42; *P* for trend = 0.022). In HRS, the corresponding incidences were 22.3, 24.2, and 31.0%, with ORs of 1.09 (95% CI: 0.84–1.42; *p* = 0.518) and 1.60 (95% CI: 0.81–3.17; *p* = 0.180) for the 2-ACE and ≥3-ACE groups, respectively. The ordinal trend was not statistically significant in HRS (OR per category increase = 1.15, 95% CI: 0.92–1.43; *P* for trend = 0.216). The ≥3-ACE subgroup in HRS was small (*n* = 42), and estimates were therefore imprecise (Supplementary Table 15).

### 3.2 LASSO-based predictor selection

**Figure 2:**
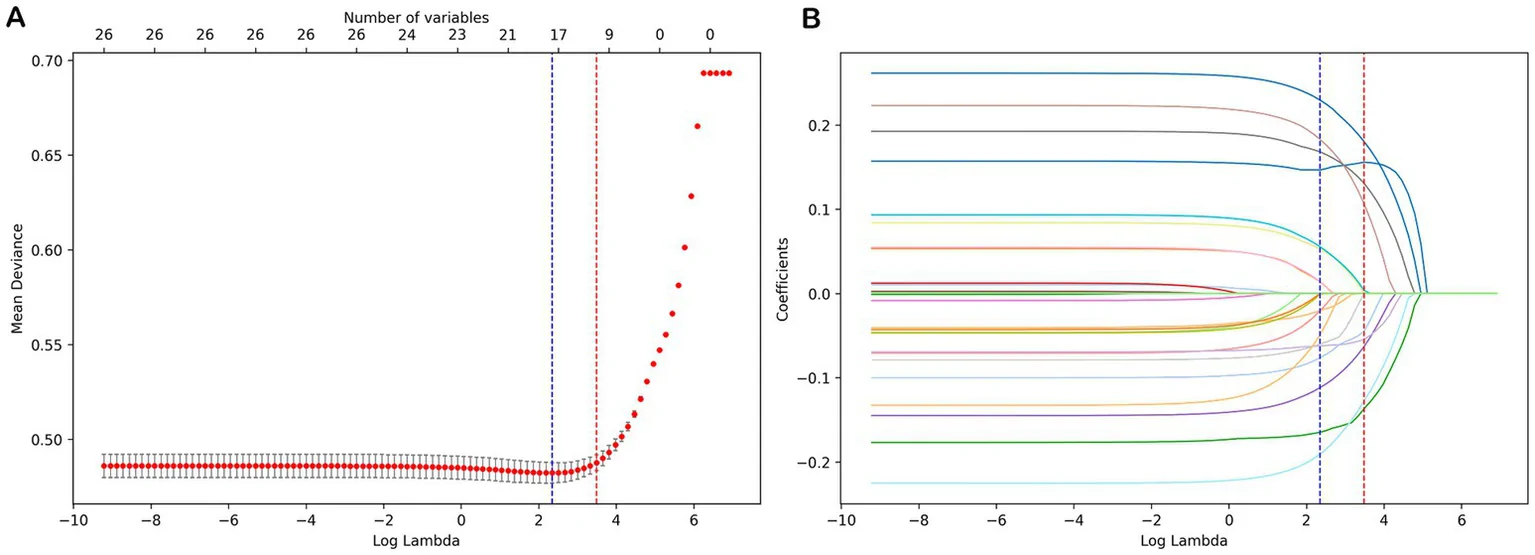
LASSO selection of baseline predictors in the ELSA development cohort. **(A)** Ten-fold cross-validation curve. Blue and red dashed lines indicate *λ*_min and the selected *λ*_1SE, respectively. **(B)** Standardized coefficient paths.

LASSO penalized logistic regression was used to select baseline predictors of incident depressive symptoms in the ELSA development cohort. The penalty parameter was determined by stratified 10-fold cross-validation. Although the minimum-deviance penalty provided the best cross-validated fit, the one-standard-error penalty was selected to obtain a more parsimonious model with potentially better generalizability (Figure 2). Across the 20 multiply imputed datasets, 11 predictors had non-zero coefficients in at least 10 datasets and were retained: falls, walking time, right-hand grip strength, arthritis, employment status, smoking status, vigorous-intensity physical activity, moderate-intensity physical activity, marital status, diabetes, and hypertension. Falls, longer walking time, arthritis, diabetes, and hypertension were positively associated with incident depressive symptoms, whereas greater right-hand grip strength and more frequent vigorous- and moderate-intensity physical activity showed negative coefficients. Detailed selection frequencies across the imputed datasets are presented in Supplementary Table 8. Self-rated health, social activity, and ADL/IADL limitations were excluded from the candidate predictor set *a priori* because of their conceptual proximity to depressive symptoms. The 11 retained predictors were subsequently used in the machine-learning model comparison and external validation.

### 3.3 Model performance and external validation

**Figure 3:**
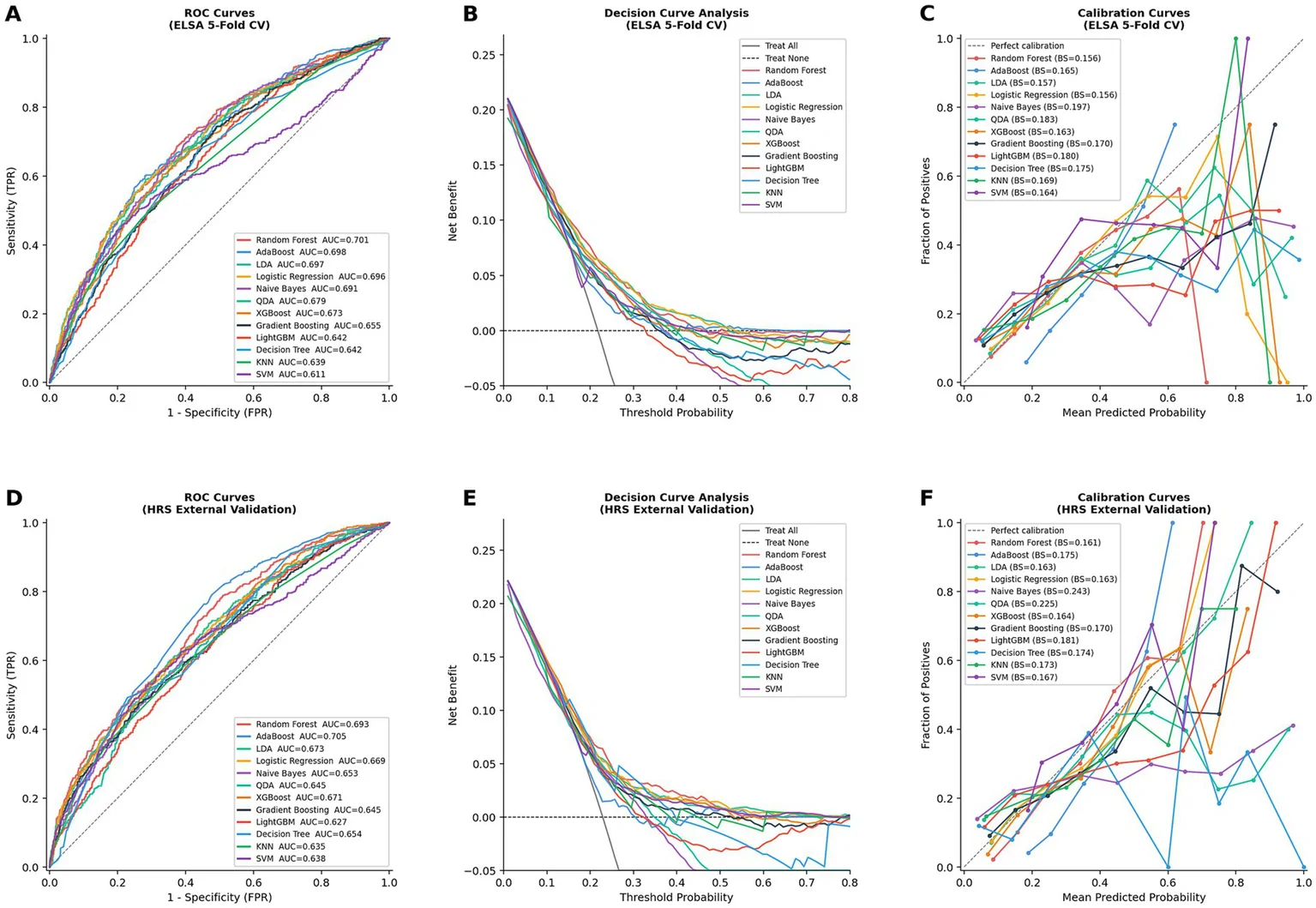
Performance comparison of machine learning models in the ELSA and HRS cohorts. **(A–C)** Model discrimination, decision curve analysis, and calibration performance in the ELSA cohort. **(D–F)** Corresponding model performance in the HRS external validation cohort.

The 12 models showed modest and broadly overlapping discrimination (Figure 3; Supplementary Table 9). In ELSA five-fold cross-validation, Random Forest achieved the highest AUC of 0.701 (95% CI: 0.672–0.727), followed by AdaBoost (0.698), LDA (0.697), and logistic regression (0.696). Random Forest also had a Brier score of 0.156. Based exclusively on its performance in the ELSA development cohort, Random Forest was selected as the final model. In HRS external validation, Random Forest retained similar discrimination, with an AUC of 0.693 (95% CI: 0.666–0.720) and a Brier score of 0.161. AdaBoost produced the highest external AUC of 0.705 (95% CI: 0.681–0.732), although its internal AUC was slightly lower than that of Random Forest and the confidence intervals overlapped. Decision curve analysis indicated modest net benefit primarily at low-to-moderate threshold probabilities, while calibration became less stable at higher predicted probabilities. At the prespecified probability threshold of 0.50, Random Forest showed low recall in both ELSA (0.089) and HRS (0.076), despite accuracies of 0.782 and 0.778, respectively. These findings indicate moderate ranking performance but limited case-detection ability at this threshold. Accordingly, the model was interpreted as an exploratory risk-prediction tool rather than a clinically deployable screening instrument.

### 3.4 SHAP interpretation of the random Forest model

**Figure 4:**
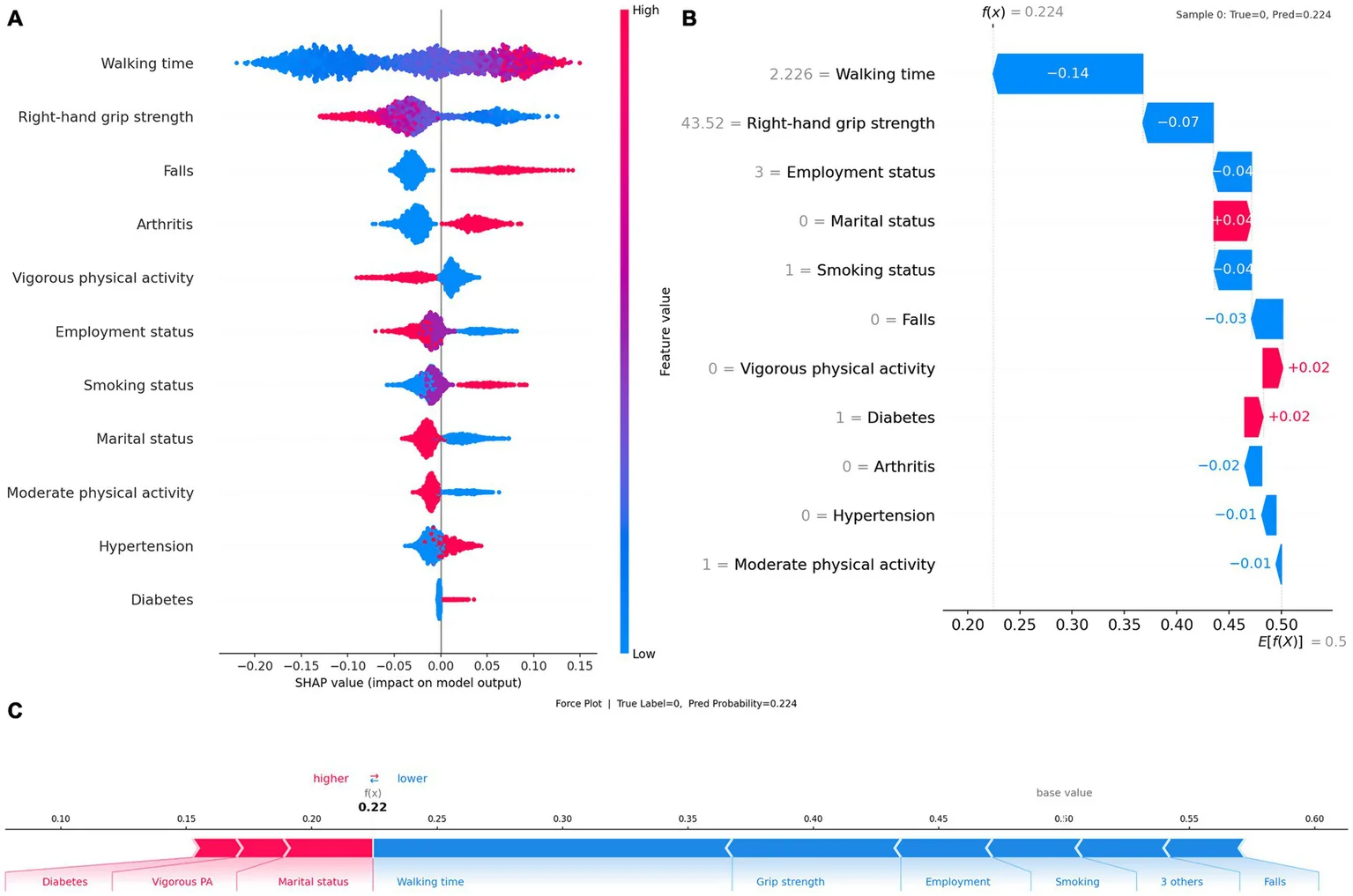
SHAP-based interpretation of the random forest model for predicted depression risk. **(A)** Global SHAP summary plot showing the relative importance and direction of key predictors. **(B,C)** Individual-level SHAP explanations illustrating how selected predictors contributed to the predicted depression risk for representative participants.

TreeSHAP analysis of the out-of-fold ELSA predictions identified walking time as the most influential predictor (mean absolute SHAP value = 0.082), followed by right-hand grip strength (0.048), falls (0.041), arthritis (0.032), and vigorous physical activity (0.020) (Figure 4A). Longer walking time, falls, and arthritis generally increased the model output, whereas greater grip strength and more frequent vigorous or moderate physical activity generally reduced it. Employment, smoking, and marital status also contributed to predictions, although their direction depended on the encoded categories. SHAP dependence plots indicated nonlinear relationships between several predictors and model output (Supplementary Figure 1). The contribution of walking time increased sharply at higher values, whereas greater grip strength was associated with progressively lower SHAP values. Falls and arthritis shifted predictions upward, while vigorous physical activity generally shifted them downward. Differences were also observed across employment-status categories. The waterfall and force plots illustrate how predictors contributed to an individual prediction (Figures 4B,C). For the illustrated participant without incident depressive symptoms, shorter walking time and greater grip strength produced the largest downward contributions, resulting in a predicted probability of 0.224. Conversely, marital status, absence of vigorous physical activity, and diabetes made smaller upward contributions. In the HRS external validation cohort, walking time remained the leading predictor across the 1-ACE and 2-ACE groups, followed by grip strength, falls, and arthritis (Supplementary Figures 2A,B). The ≥3-ACE group showed greater variability in predictor rankings because of its small sample size (*n* = 42; Supplementary Figure 2C). These ACE-stratified SHAP patterns were considered exploratory and were not interpreted as evidence of effect modification.

### 3.5 Activity-function transition profiles and incident depressive symptoms

**Figure 5:**
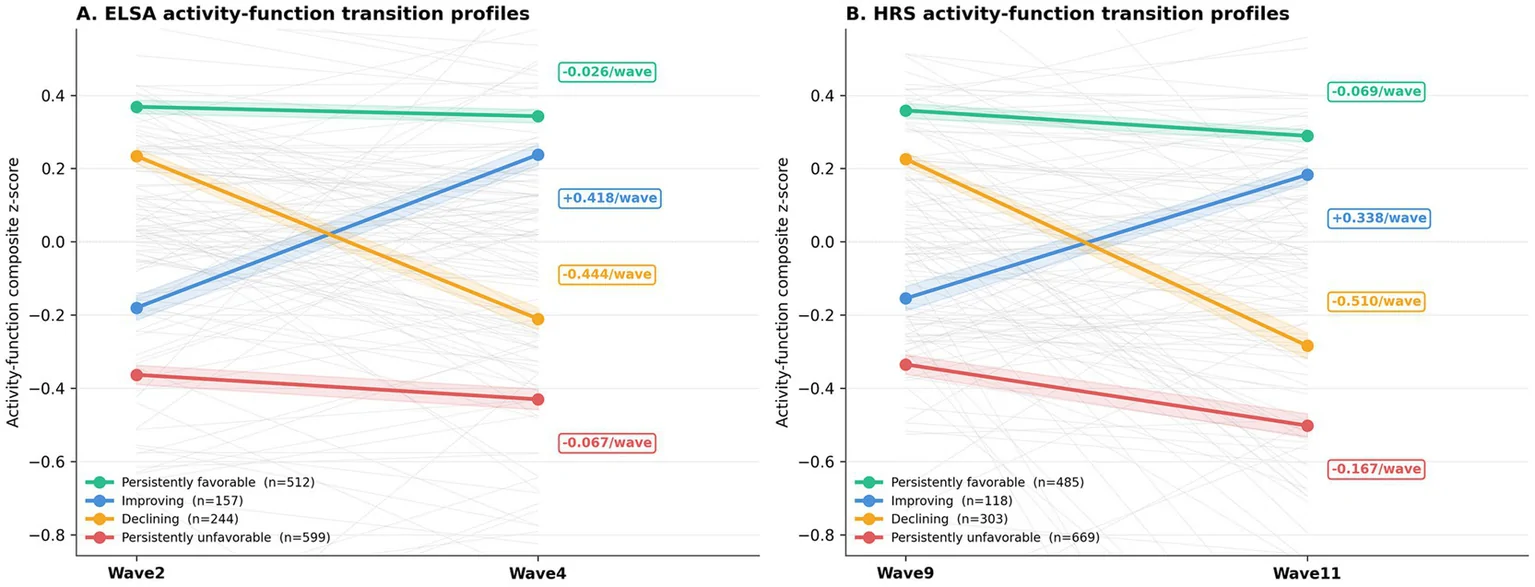
Pre-outcome activity-function transition profiles in ELSA and HRS. Activity-function composite scores are shown from Waves 2 to 4 in ELSA **(A)** and Waves 9 to 11 in HRS **(B)**.

Given the *a priori* focus on activity and physical function, and the prominent SHAP contributions of walking time and moderate- and vigorous-intensity physical activity, we further examined whether pre-outcome changes in these indicators were associated with actual incident depressive symptoms, independently of model-predicted risk. Four activity-function transition profiles were identified in both cohorts (Figure 5). In ELSA, incident depressive symptoms occurred in 8.0% of the persistently favorable group, 14.0% of the improving group, 12.7% of the declining group, and 21.9% of the persistently unfavorable group. The corresponding incidences in HRS were 8.5, 7.6, 17.2, and 18.7%, respectively (Table 1). After adjustment for age, sex, and education, the persistently unfavorable profile was associated with higher odds of incident depressive symptoms in both ELSA (OR = 2.59, 95% CI: 1.69–3.97) and HRS (OR = 2.34, 95% CI: 1.54–3.55). The declining profile was also associated with higher odds in HRS (OR = 2.19, 95% CI: 1.39–3.44), but not in ELSA (OR = 1.48, 95% CI: 0.89–2.47). The improving profile was not significantly associated with the outcome after adjustment in either cohort. Complete unadjusted and adjusted estimates are presented in Supplementary Table 10. ACE-stratified analyses showed that the persistently unfavorable profile had the highest incidence in both ACE-burden groups in ELSA, whereas the declining and persistently unfavorable profiles generally showed higher incidence in HRS (Supplementary Figures 3, 4). However, formal interaction tests provided no evidence that ACE burden modified the profile–outcome associations under either the binary or three-level ACE classification (all *p* for interaction >0.05; Supplementary Table 12). Therefore, the stratified differences were interpreted descriptively rather than as evidence of effect modification. In the full-sample sensitivity analysis, the persistently unfavorable profile remained significantly associated with subsequent depressive symptoms in both cohorts, and the declining profile remained significant in HRS. Additional associations emerged in ELSA, indicating some sensitivity to the exclusion of participants with baseline depressive symptoms (Supplementary Table 11).

**Table 1:** Association between pre-outcome activity-function transition profiles and incident depression in ELSA and HRS.

Cohort	Profile definition	Outcome	Profile	*N*	Incident depression	Adjusted OR	95%CI
ELSA	Wave2 → Wave4	Wave 6 incident depression	Persistently favorable	512	8%	1	Reference
			Improving	157	14%	1.68	0.96–2.95
			Declining	244	12.7%	1.48	0.89–2.47
			Persistently unfavorable	599	21.9%	2.59	1.69–3.97
HRS	Wave9 → Wave11	Wave 13 incident depression	Persistently favorable	485	8.5%	1	Reference
			Improving	118	7.6%	0.84	0.39–1.79
			Declining	303	17.2%	2.19	1.39–3.44
			Persistently unfavorable	669	18.7%	2.34	1.54–3.55

## 4 Discussion

The present multi-cohort study developed and externally validated an interpretable prediction framework for incident depressive symptoms among middle-aged and older adults with reported adverse childhood experiences. Several findings merit emphasis. First, incident depressive symptoms were consistently more common among participants with lower socioeconomic position, poorer health status, lower physical activity, and worse physical function at baseline. Second, after LASSO-based feature selection and comparison of 12 candidate classifiers, the random forest model showed moderate discrimination in ELSA and retained similar performance in the independent HRS cohort. Third, SHAP analysis indicated that walking time, grip strength, falls, arthritis, and vigorous physical activity were the most influential contributors to model-predicted risk. Finally, the activity-function transition analysis showed that participants with persistently unfavorable activity-function profiles had higher odds of subsequent depressive symptoms in both cohorts, whereas evidence for the improving profile and for ACE-burden interaction was limited. Together, these findings suggest that physical-performance and activity-related indicators provide meaningful information for exploratory characterization of subsequent depressive-symptom risk among ACE-exposed middle-aged and older adults, while also underscoring that the model remains a research framework rather than a tool ready for direct clinical deployment.

These results are broadly consistent with life-course evidence linking ACE exposure with later-life mental health vulnerability. Prior studies have shown that childhood adversity is associated with depressive symptoms, chronic disease burden, pain, functional limitations, and other health outcomes in adulthood and older age (28–30). The present study extends this literature by focusing specifically on individuals with reported ACE exposure, by emphasizing incident depressive symptom status over follow-up, and by integrating conventional feature selection, machine-learning prediction, external validation, and interpretable model explanation. This design shifts attention from single-factor associations toward a multivariable risk-pattern framework within a population that remains heterogeneous despite shared exposure to childhood adversity.

The prominence of walking time, grip strength, falls, arthritis, and physical activity in the prediction model also agrees with previous literature on late-life depression, physical activity, and functional health (31, 32). These variables may capture several overlapping dimensions of vulnerability, including mobility reserve, pain or musculoskeletal burden, chronic disease accumulation, independence in daily life, and opportunities for social engagement (33, 34). In this context, the SHAP findings are clinically interpretable: longer walking time and fall history increased predicted risk, whereas greater grip strength and more frequent vigorous or moderate physical activity generally lowered model-predicted risk. Importantly, these patterns should not be interpreted as evidence that changing any single factor would necessarily alter depression risk. Instead, the findings indicate that physical function and activity-related measures are informative markers within a broader risk profile among ACE-exposed middle-aged and older adults.

The activity-function transition analysis provides additional information on changes in activity-function status between the two pre-outcome assessments. Participants who remained in the persistently unfavorable profile had the highest or among the highest incidence of depressive symptoms in both ELSA and HRS, and this profile remained associated with the outcome after adjustment for age, sex, and education (35). The declining profile was associated with higher odds in HRS but not in ELSA, whereas the improving profile was not statistically significant after adjustment in either cohort. This pattern suggests that sustained unfavorable activity-function status may carry more stable predictive information than short-term improvement defined by a two-wave composite (36, 37). Differences between cohorts may reflect measurement heterogeneity, sample composition, health-system and social-context differences, or limited statistical power within specific transition groups. The absence of statistically significant interaction by ACE burden further suggests that, within this ACE-exposed sample, activity-function profiles were not clearly different in their associations across ACE strata; however, the small size of the highest ACE-burden subgroup means that these subgroup findings should remain descriptive. These associations should not be interpreted as evidence that improving activity-function status would itself reduce depressive-symptom incidence, because residual confounding and reverse causation cannot be excluded in this observational design.

The predictive performance of the machine-learning models should be interpreted with appropriate caution. The random forest model achieved an AUC of 0.701 in ELSA cross-validation and 0.693 in HRS external validation, indicating moderate ranking ability. The similar internal and external performance is encouraging because prediction models often lose accuracy when transported across cohorts, particularly when predictor definitions, sampling frames, and outcome measurement contexts differ. At the same time, the low recall at the prespecified 0.50 threshold shows that the model would miss many participants who subsequently report depressive symptoms if used with that threshold. The decision-curve and calibration findings likewise indicate that potential net benefit was most apparent at low-to-moderate threshold probabilities and that calibration was less stable at higher predicted probabilities. Thus, the model is best viewed as an interpretable research tool for exploratory risk characterization and hypothesis generation, not as a stand-alone screening or clinical decision-making instrument.

Compared with traditional regression-only approaches, the study contributes methodologically by combining parsimonious feature selection, multiple imputation within validation procedures, external validation in an independent aging cohort, and SHAP-based interpretation. This design helps address two common limitations in depression prediction studies: overreliance on internal validation and limited transparency of complex algorithms. Although AdaBoost yielded a slightly higher external AUC, the confidence intervals overlapped and the final random forest model was selected according to the development-cohort performance before external validation, reducing the risk of selecting a model based on the external test cohort. The SHAP analysis further made the model outputs more interpretable by identifying both global predictor importance and participant-level contributions, consistent with the growing emphasis on explainable artificial intelligence in health prediction research (38, 39).

The findings may have value primarily for aging research and future development of depression risk-prediction approaches. Because the most influential predictors are commonly collected in large aging cohorts and many clinical or community assessments, the framework may provide a basis for further investigation of multivariable risk patterns in ACE-exposed populations. The transition-profile analysis may also help researchers distinguish participants with unfavorable status at both assessments from those showing different two-point transition patterns. Any practical use, however, would require prospective validation, context-specific calibration, and careful assessment of fairness, acceptability, and clinical workflow fit.

Several limitations should be acknowledged. First, ACE exposure was assessed retrospectively and harmonized across two cohorts with different questionnaire structures. The ≥1-ACE definition facilitated identification of a comparable ACE-exposed target population but necessarily reduced information on adversity subtype, timing, severity, and cumulative exposure. Although secondary analyses examined cumulative ACE-burden categories and identified a significant ordinal trend in ELSA, this pattern was not statistically confirmed in HRS, where the highest-burden subgroup was small. Differences in cohort-specific ACE items also limit direct interpretation of ACE counts as equivalent quantitative doses across cohorts. Second, depressive symptoms were measured using the CES-D-8 rather than diagnostic interviews, so the outcome should be interpreted as incident depressive symptoms rather than clinically diagnosed depression. Third, physical activity was based on cohort-specific self-report frequency measures, and the activity-function composite relied on only two pre-outcome time points and median-based dichotomization. Fourth, although the study preserved temporal ordering and excluded participants with depressive symptoms at baseline, the observational design does not establish causality. Residual confounding and reverse causation remain possible because adult-life adversity, pain severity, medication use, sleep, cognition, social support quality, and access to care were not fully captured. Accordingly, associations involving physical activity, physical performance, and subsequent depressive symptoms should not be interpreted as evidence that modifying these factors would alter outcome risk. Fifth, predictor missingness was substantial and was associated with several measured baseline characteristics. In particular, some missingness reflected age-dependent survey administration patterns, such that complete-case analysis would have resulted in a highly selected subset of participants. Multiple imputation was therefore used to retain eligible participants and incorporate available information across predictors. Nevertheless, multiple imputation relies on assumptions regarding the missing-data mechanism and cannot eliminate bias related to unmeasured determinants of missingness. In addition, attrition over the approximately 8-year follow-up may have introduced further selection bias, particularly if participants unavailable at the final outcome assessment differed systematically from those retained. This potential attrition-related bias cannot be fully excluded. Sixth, external validation was conducted in HRS only; generalizability to non-Western populations, clinical samples, and settings with different ACE profiles requires further evaluation.

Future work could improve the evidence base in several directions. More detailed ACE characterization may clarify whether specific adversity domains or cumulative adversity patterns contribute additional predictive information beyond the binary ACE-exposed definition. Longer and denser repeated measurements could support latent trajectory modelling or sequence-based approaches that better capture changes in activity, function, chronic disease, and social participation over time. Model development could also incorporate calibration updating, threshold optimization, fairness analyses, and decision-analytic evaluation in the populations where the model may be used. Finally, prospective studies with harmonized measurements, objective activity or mobility data, and richer psychosocial variables would help determine whether the activity-function predictors identified here remain robust markers of future depressive-symptom risk across diverse aging contexts.

## 5 Conclusion

In conclusion, this study provides externally validated and interpretable evidence that physical activity and physical function indicators are important components of depression risk prediction among ACE-exposed middle-aged and older adults. By integrating longitudinal cohort data, machine-learning model comparison, external validation, SHAP-based interpretation, and analysis of two-point activity-function transitions, this study offers a transparent framework for characterizing risk heterogeneity in a vulnerable aging population. Given the observational design and moderate predictive performance, these findings should be interpreted as supporting exploratory risk characterization and future model refinement rather than clinical screening, individual decision making, causal inference, or direct behavioral recommendations.

## Data Availability

Publicly available datasets were analyzed in this study. This data can be found here: The datasets analyzed in this study are available to registered or qualified researchers through their official repositories. Health and Retirement Study (HRS) data are available from the HRS Data Portal: https://hrsdata.isr.umich.edu/. English Longitudinal Study of aging (ELSA) data are available through the United Kingdom Data Service: https://beta.ukdataservice.ac.uk/datacatalogue/series/series?id=200011.

## References

[1] Zhu L Wang Y Li J Zhou H Li N Wang Y. Depressive symptoms and all-cause mortality among middle-aged and older people in China and associations with chronic diseases. Front Public Health. (2024) 12:1381273. doi: 10.3389/fpubh.2024.1381273, 38841667 PMC11151855

[2] Ni Z Zhu X Tian K Chen Q Yang Y Xie S. Depressive symptoms of older adults with chronic diseases: the mediating roles of activities of daily living and economic burden of diseases. Front Psychol. (2024) 15:1387677. doi: 10.3389/fpsyg.2024.1387677, 39015326 PMC11249773

[3] Lee SL Pearce E Ajnakina O Johnson S Lewis G Mann F . The association between loneliness and depressive symptoms among adults aged 50 years and older: a 12-year population-based cohort study. Lancet Psychiatry. (2021) 8:48–57. doi: 10.1016/S2215-0366(20)30383-7, 33181096 PMC8009277

[4] Seo M-W. Association of health literacy with depressive and anxiety symptoms in Korean adults: a dose–response analysis. BMC Psychol. (2026) 14:992. doi: 10.1186/s40359-026-04765-2, 42129934 PMC13340370

[5] Liu L Tang L Dai M Ding X Wu L Ke X . Transcultural prediction model for late-life depression based on multi-cohort machine learning and explainable AI. J Affect Disord. (2026) 392:120169. doi: 10.1016/j.jad.2025.120169, 40882851

[6] Yu P Wang X Liu J Luo H Yi Y. Adverse childhood experiences, marital status and depressive symptoms in later life among the Chinese middle-aged and older adults: the mediating role of marital status. BMC Public Health. (2024) 24:2246. doi: 10.1186/s12889-024-19787-x, 39160540 PMC11331659

[7] Taylor K Demakakos P. Adverse childhood experiences and trajectories of multimorbidity in individuals aged over 50: evidence from the English longitudinal study of ageing. Child Abuse Negl. (2024) 149:106653. doi: 10.1016/j.chiabu.2024.106653, 38277873

[8] Li Q Chen L Wang R. Adverse childhood experiences and late-life depression in older Chinese adults: the mediating roles of sleep disorders and chronic diseases. BMC Geriatr. (2025) 25:1076. doi: 10.1186/s12877-025-06740-9, 41469581 PMC12755008

[9] Richardson S Carr E Netuveli G Sacker A. Adverse events over the life course and later-life wellbeing and depressive symptoms in older people. Int Psychogeriatr. (2023) 35:243–57. doi: 10.1017/S1041610220003373, 33050971

[10] Huang Y Dong Y Wang Y Wang G Lu Z Wang Q. Associations between adverse childhood experiences and physical activity domains among middle-aged and older adults: a national analysis. BMC Public Health. (2026) 26:769. doi: 10.1186/s12889-026-26419-z, 41629898 PMC12955266

[11] Mengist B Lotfaliany M Pasco JA Agustini B Berk M Williams LJ . Gait speed, handgrip strength, and their combination, and risk of depression in later life: evidence from a prospective study of community-dwelling older adults. J Affect Disord. (2025) 369:218–26. doi: 10.1016/j.jad.2024.09.155, 39353510

[12] Seo M-W. Health literacy domains and socioeconomic inequalities in leisure-time physical activity among Korean adults. Front Public Health. (2026) 14:1846028. doi: 10.3389/fpubh.2026.1846028, 42293620 PMC13253458

[13] Wang S Wu Z Guo Z Wang F Liu B. The impact of physical and mind exercise on functional disability in activities of daily living among the oldest old. BMC Public Health. (2025) 25:1813. doi: 10.1186/s12889-025-22968-x, 40380313 PMC12082957

[14] Wang Y Wang X Zhao L Jones K. A case for the use of deep learning algorithms for individual and population level assessments of mental health disorders: predicting depression among China’s elderly. J Affect Disord. (2025) 369:329–37. doi: 10.1016/j.jad.2024.09.147, 39321977

[15] Long Q Li J Zhao N Guo R Tang S. Deep learning for depression prediction in older adults: a retrospective cohort study from CHARLS (2011-2020) with independent cohort validation in CLHLS (2008-2018). J Affect Disord. (2026) 400:121206. doi: 10.1016/j.jad.2026.121206, 41554486

[16] Steptoe A Breeze E Banks J Nazroo J. Cohort profile: the English longitudinal study of ageing. Int J Epidemiol. (2013) 42:1640–8. doi: 10.1093/ije/dys168, 23143611 PMC3900867

[17] Sonnega A Faul JD Ofstedal MB Langa KM Phillips JW Weir DR. Cohort profile: the health and retirement study (HRS). Int J Epidemiol. (2014) 43:576–85. doi: 10.1093/ije/dyu067, 24671021 PMC3997380

[18] Lin Z Sim Y-J Zhang X. Predicting the risk of depression and cognitive impairment in older adults based on physical activity trajectories: a multi-cohort study using attention mechanisms and machine learning. Ment Health Phys Act. (2026) 30:100777. doi: 10.1016/j.mhpa.2026.100777

[19] Ding Y Duan M Shen J Xu L Ma Y He D . Associations of physical frailty, depression and their interaction with incident all-cause dementia among older adults: evidence from three prospective cohorts. Gen Psychol. (2025) 38:e102172. doi: 10.1136/gpsych-2025-102172 PMC12718578 41431467

[20] Zhang Z Liu Z Ye F Mo D Shi Y Tao P . Adverse childhood experiences and hypertension jointly increase cardiovascular risk: evidence from three national aging cohorts. BMC Public Health. (2026) 26. doi: 10.1186/s12889-026-27061-5, 41862855 PMC13126772

[21] Carr AL Massou E Kelly MP Ford JA. Mediating pathways that link adverse childhood experiences with cardiovascular disease. Public Health. (2024) 227:78–85. doi: 10.1016/j.puhe.2023.11.027, 38134567

[22] Zheng G Fang Z Zhou B He F Zhang H Xiao H . Association between adverse childhood experiences and long-term blood pressure variability: insights from a pooled analysis. J Affect Disord. (2025) 384:118–24. doi: 10.1016/j.jad.2025.05.039, 40345441

[23] Garin N Koyanagi A Chatterji S Tyrovolas S Olaya B Leonardi M . Global multimorbidity patterns: a cross-sectional, population-based, multi-country study. J Gerontol A Biol Sci Med Sci. (2016) 71:205–14. doi: 10.1093/gerona/glv128, 26419978 PMC5864156

[24] Lu C Wan S Liu Z. Determinants of depressive symptoms in multinational middle-aged and older adults. NPJ Digit Med. (2025) 8:501. doi: 10.1038/s41746-025-01905-7, 40759736 PMC12321985

[25] Demakakos P Hamer M Stamatakis E Steptoe A. Low-intensity physical activity is associated with reduced risk of incident type 2 diabetes in older adults: evidence from the English longitudinal study of ageing. Diabetologia. (2010) 53:1877–85. doi: 10.1007/s00125-010-1785-x, 20495973

[26] Hamer M Muniz G Demakakos P. Physical activity and trajectories in cognitive function: English longitudinal study of ageing. J Epidemiol Community Health. (2018) 72:477–83. doi: 10.1136/jech-2017-210228, 29434025 PMC5977988

[27] ShangGuan Y Ji K Lin Z He C Sim Y-J Liu H . Multidimensional dietary assessment and interpretable machine learning models predict the risk of prediabetes/diabetes and osteoporosis comorbidity in older adults. Front Nutr. (2025) 12:1666477. doi: 10.3389/fnut.2025.1666477, 41334340 PMC12667436

[28] Hughes K Bellis MA Hardcastle KA Sethi D Butchart A Mikton C . The effect of multiple adverse childhood experiences on health: a systematic review and meta-analysis. Lancet Public Health. (2017) 2:e356–66. doi: 10.1016/S2468-2667(17)30118-4, 29253477

[29] Zhang N Yao Y Li L Sun M Zhou B Fu H . Deprivation-related adverse childhood experiences and cognitive function among older adults: mediating role of depression symptoms. Child Abuse Negl. (2024) 158:107088. doi: 10.1016/j.chiabu.2024.107088, 39406057

[30] D’Arcy-Bewick S Turiano N Sutin AR Terracciano A O’Súilleabháin PS. Adverse childhood experiences and all-cause mortality risk in adulthood. Child Abuse Negl. (2023) 144:106386. doi: 10.1016/j.chiabu.2023.106386 37542995

[31] Pearce M Garcia L Abbas A Strain T Schuch FB Golubic R . Association between physical activity and risk of depression: a systematic review and Meta-analysis. JAMA Psychiatry. (2022) 79:550–9. doi: 10.1001/jamapsychiatry.2022.0609, 35416941 PMC9008579

[32] Sun A Liu Z. Association between relative grip strength and depression among U.S. middle-aged and older adults: results from the NHANES database. Front Public Health. (2024) 12:1416804. doi: 10.3389/fpubh.2024.1416804, 39135921 PMC11317278

[33] López-Bueno R Calatayud J Andersen LL Casaña J Koyanagi A Cruz B . Dose–response association of handgrip strength and risk of depression: a longitudinal study of 115 601 older adults from 24 countries. Br J Psychiatry. (2023) 222:135–42. doi: 10.1192/bjp.2022.178, 36464972 PMC9929711

[34] Li Y Su S Luo B Wang J Liao S. Physical activity and depressive symptoms among community-dwelling older adults in the COVID-19 pandemic era: a three-wave cross-lagged study. Int J Disaster Risk Reduct. (2022) 70:102793. doi: 10.1016/j.ijdrr.2022.102793, 35036301 PMC8744411

[35] Gao Y Jia Z Zhao L Han S. The effect of activity participation in middle-aged and older people on the trajectory of depression in later life: National Cohort Study. JMIR Public Health Surveill. (2023) 9:e44682. doi: 10.2196/44682, 36951932 PMC10131905

[36] Mengist B Lotfaliany M Pasco JA Agustini B Berk M Orchard SG . Temporal dynamics and bidirectional longitudinal association between physical function and depressive symptoms in older adults. J Cachexia Sarcopenia Muscle. (2026) 17:e70223. doi: 10.1002/jcsm.70223, 41616761 PMC12858256

[37] Seo M-W Eum Y Jung HC. Leisure time physical activity: a protective factor against metabolic syndrome development. BMC Public Health. (2023) 23:2449. doi: 10.1186/s12889-023-17340-w, 38062414 PMC10701969

[38] Lin Z Sim Y-J Zhang C Wu K Sun Z ShangGuan Y . Sarcopenia risk assessment among physically inactive middle-aged and older adults: interpretable machine-learning models in UK and US cohorts. Prim Health Care Res Dev. (2026) 27:e71. doi: 10.1017/S1463423626101364

[39] Shangguan Y Lin Z Sim Y-J Wu K Chu Y Huang K . Development and external validation of an interpretable machine learning model for obesity-depression comorbidity in Korean and US adults. Int J Public Health. (2026) 71:1609153. doi: 10.3389/ijph.2026.1609153, 42292917 PMC13254829

