# Supplementary material for: Activity-function transitions and interpretable machine learning for predicting incident depressive symptoms among ACE-exposed middle-aged and older adults: a multi-cohort study
Source: Front Public Health. 2026 Sep 3;14:1924836. doi: 10.3389/fpubh.2026.1924836 (PMC13584956; doi:10.3389/fpubh.2026.1924836)
Supplement: Supplementary file 1 [file Supplementary_file_1.DOCX]

**Supplementary Material**

**Supplementary Figure 1.** SHAP Dependence Plots for Key Predictors in the Random Forest Model

**Supplementary Figure 2.** Exploratory SHAP distributions of predictors stratified by ACE burden in the HRS external validation cohort

**Supplementary Figure 3.** ACE-stratified incidence of depression and associations with activity-function transition profiles in ELSA

**Supplementary Figure 4.** ACE-stratified incidence of depression and associations with activity-function transition profiles in HRS

**Supplementary Table 1.** Variable definitions and harmonization details

**Supplementary Table 2.** Baseline characteristics of ELSA cohort participants (Wave 2)

**Supplementary Table 3.** Baseline characteristics of ELSA cohort participants (Wave 4)

**Supplementary Table 4.** Baseline characteristics of ELSA cohort participants (Wave 6)

**Supplementary Table 5.** Baseline characteristics of HRS cohort participants (Wave 9)

**Supplementary Table 6.** Baseline characteristics of HRS cohort participants (Wave 11)

**Supplementary Table 7.** Baseline characteristics of HRS cohort participants (Wave 13)

**Supplementary Table 8.** Stability of baseline predictor selection across 20 multiply imputed datasets in the ELSA development cohort

**Supplementary Table 9.** Comprehensive Predictive Performance of 12 Machine Learning Models in ELSA Internal Validation and HRS External Validation

**Supplementary Table 10.** Unadjusted and adjusted associations between activity-function transition profiles and incident depression

**Supplementary Table 11.** Sensitivity analysis comparing the primary incident-depression sample with the full analytical sample

**Supplementary Table 12.** Interaction tests between ACE burden and activity-function transition profiles for incident depression in ELSA and HRS

**Supplementary Table 13.** Baseline characteristics according to predictor-data completeness in ELSA

**Supplementary Table 14.** Baseline characteristics according to predictor-data completeness in HRS

**Supplementary Table 15.** Association between cumulative ACE burden and incident depressive symptoms in ELSA and HRS

**Supplementary Figure 1.** SHAP Dependence Plots for Key Predictors in the Random Forest Model

**Supplementary Figure 2.** Exploratory SHAP distributions of predictors stratified by ACE burden in the HRS external validation cohort

**Supplementary Figure 3.** ACE-stratified incidence of depression and associations with activity-function transition profiles in ELSA

**Supplementary Figure 4.** ACE-stratified incidence of depression and associations with activity-function transition profiles in HRS

**Supplementary Table 1.** Variable definitions and harmonization details

| Variable Type | Variable Name | Variable Value | Handling of Variable | HRS | ELSA |
| --- | --- | --- | --- | --- | --- |
| **Dependent Variable** | Depressive symptoms | No = 0,  Yes = 1 | No = 0,  Yes = 1 | CESD-8 score less than 3 indicates no depressive symptoms risk = 0 | CESD-8 score less than 3 indicates no depressive symptoms risk = 0 |
| **Population Selection Variable** | Adverse childhood experiences (ACEs) | No = 0,  Yes = 1 | No = 0,  Yes = 1 | ACEs were assessed using 12 self-reported items: physical abuse; parental drink and drug problems; separation from father or mother; parental separation or divorce; adverse parental relationships; residing in a foster home, orphanage, or boarding school; childhood poverty; emotional neglect; repeat year of school; trouble with police; death of a parent; and death of a sibling. | ACEs were assessed using 12 self-reported items, including physical abuse, household substance abuse or mental health problems, parental arguments, separation from mother, parents’ divorce, death of a parent, sexual assault, physical assault, care home/foster care experience, institutionalization, low maternal bonding, and low paternal bonding. |
|  | Diabetes | Self-reported physician diagnosis; No = 0, Yes = 1 | Self-reported physician diagnosis; No = 0, Yes = 1 | Consistent | Consistent |
|  | Age | 50 ≤ age < 60 = 1, 60 ≤ age < 90 = 2, age ≥ 90 = 3 | 50 ≤ age < 60 = 1, 60 ≤ age < 90 = 2, age ≥ 90 = 3 | Consistent | Consistent |
|  | Gender | Female = 2, Male = 1 | Female = 2, Male = 1 | Consistent | Consistent |
|  | Marital Status | Never married, separated, divorced, widowed, married but absent spouse = 0, Married = 1 | Never married, separated, divorced, widowed, married but absent spouse = 0, Married = 1 | Never married, separated, divorced, widowed, or married but spouse absent = 0 | Never married, separated, divorced, or widowed = 0 |
|  | Education Level | Less than middle school = 1, High school = 2, Higher education = 3 | Less than middle school = 1, High school = 2, Higher education = 3 | Consistent | Consistent |
|  | Employment Status | Unemployed = 1, Retired = 2, Working = 3 | Unemployed = 1, Retired = 2, Working = 3 | Unemployed = 1, Retired, other status and retired = 2, Employed = 3 | Unemployed, permanently ill or disabled, homemaker = 1, Retired = 2, Employed or self-employed = 3 |
| **Lifestyle Factors** | Smoking | Never smoked = 1, Former smoker = 2, Current smoker = 3 | Never smoked = 1, Former smoker = 2, Current smoker = 3 | Consistent | Consistent |
|  | Drinking | Never drink = 0, Currently or formerly drinks = 1 | Not drinking now = 0, Drinking now = 1 | Currently not drinking = 0, Currently drinking = 1 | Does not drink weekly = 0, Drinks weekly or within the past 7 days = 1 |
|  | HighIntensityPA | Low frequency = 0, High frequency = 1 | Frequency scale: 1=Every day, 2=>1/week, 3=1/week, 4=1-3/mon, 5=Never. Recoded as: 0 = Low frequency (Options 4-5); 1 = High frequency (Options 1-3) | Consistent | Consistent |
|  | MediumIntensityPA | Low frequency = 0, High frequency = 1 | Frequency scale: 1=Every day, 2=>1/week, 3=1/week, 4=1-3/mon, 5=Never. Recoded as: 0 = Low frequency (Options 4-5); 1 = High frequency (Options 1-3) | Consistent | Consistent |
|  | LowIntensityPA | Low frequency = 0, High frequency = 1 | Frequency scale: 1=Every day, 2=>1/week, 3=1/week, 4=1-3/mon, 5=Never. Recoded as: 0 = Low frequency (Options 4-5); 1 = High frequency (Options 1-3) | Consistent | Consistent |
| **Health Status** | Self-rated Health | Poor = 1, Fair = 2, Good, Very Good = 3 | Poor = 1, Fair = 2, Good, Very Good = 3 | Poor = 1, Fair = 2, Good, very good, excellent = 3 | Poor = 1, Fair = 2, Good, very good, excellent = 3 |
|  | ADL | No = 0, Yes = 1 | No difficulty in basic ADLs = 0, Difficulty in one or more = 1 | No difficulty in dressing, bathing, eating, getting in and out of bed, using toilet, or walking around = 0, Difficulty in any of the above tasks = 1 | No difficulty in dressing, bathing, eating, getting in and out of bed, using toilet, or walking around = 0, Difficulty in any of the above tasks = 1 |
|  | IADL | No = 0, Yes = 1 | No difficulty in instrumental ADLs = 0, Difficulty in one or more = 1 | No difficulty in cleaning, cooking, shopping, calling, taking medications, or managing finances = 0, Difficulty in any of the above tasks = 1 | No difficulty in using phone, taking medications, managing finances, shopping, cooking, using maps, working around the house and garden, leaving home alone, or using transportation = 0, Difficulty in any of the above tasks = 1 |
|  | Visual.impairment | No = 0, Yes = 1 |  | Visual impairment is diagnosed when both distance and near vision scores are 5 Poor and 6 Poor. | Visual impairment is defined as a visual acuity of 5 Poor in both distance and near vision. |
|  | Falls | No = 0, Yes = 1 | Reported falling in the last two years = 1, No falls = 0 | Consistent | Consistent |
|  | Hypertension | No = 0, Yes = 1 | Self-reported doctor diagnosis of hypertension = 1, No = 0 | Consistent | Consistent |
|  | Stroke | No = 0, Yes = 1 | Self-reported doctor diagnosis of stroke = 1, No = 0 | Consistent | Consistent |
|  | Cancer | No = 0, Yes = 1 | Self-reported doctor diagnosis of cancer (excluding minor skin cancers) = 1, No = 0 | Consistent | Consistent |
|  | Arthritis | No = 0, Yes = 1 | Self-reported doctor diagnosis of arthritis = 1, No = 0 | Consistent | Consistent |
| Socioeconomic Status | Pension | No = 0, Yes = 1 | Currently receiving pension benefits = 1, Not receiving = 0 | Consistent | Consistent |
|  | Social Activity | No = 0, Yes = 1 | Participate in community activities = 1,  Did not participate in social activities = 0 | Consistent | Consistent |

**Supplementary Table 2.** Baseline characteristics of ELSA cohort participants (Wave 2)

| Variable | Overall | Non_Depressed | Depressed | P_Value |
| --- | --- | --- | --- | --- |
| Gender | N=1920 | N=1512 | N=408 | <0.001 |
| Male | 832 (43.3%) | 707 (46.8%) | 125 (30.6%) | |
| Female | 1088 (56.7%) | 805 (53.2%) | 283 (69.4%) | |
| Education | N=1920 | N=1512 | N=408 | <0.001 |
| Less than middle school | 826 (43.0%) | 599 (39.6%) | 227 (55.6%) | |
| High school | 343 (17.9%) | 269 (17.8%) | 74 (18.1%) |  |
| Higher education | 751 (39.1%) | 644 (42.6%) | 107 (26.2%) | |
| Adverse Childhood Experiences | N=1920 | N=1512 | N=408 | <0.001 |
| Yes | 1920 (100.0%) | 1512 (100.0%) | 408 (100.0%) | |
| Wave2HighIntensityPA | N=1920 | N=1512 | N=408 | <0.001 |
| Low frequency | 1278 (66.6%) | 964 (63.8%) | 314 (77.0%) | |
| High frequency | 642 (33.4%) | 548 (36.2%) | 94 (23.0%) |  |
| Wave2MediumIntensityPA | N=1920 | N=1512 | N=408 | <0.001 |
| Low frequency | 390 (20.3%) | 246 (16.3%) | 144 (35.3%) | |
| High frequency | 1530 (79.7%) | 1266 (83.7%) | 264 (64.7%) | |
| Wave2LowIntensityPA | N=1920 | N=1512 | N=408 | <0.001 |
| Low frequency | 187 (9.7%) | 126 (8.3%) | 61 (15.0%) |  |
| High frequency | 1733 (90.3%) | 1386 (91.7%) | 347 (85.0%) | |
| Wave2Smoking | N=1920 | N=1512 | N=408 | <0.001 |
| Never smoked | 696 (36.2%) | 558 (36.9%) | 138 (33.8%) | |
| Former smoker | 961 (50.1%) | 775 (51.3%) | 186 (45.6%) | |
| Current smoker | 263 (13.7%) | 179 (11.8%) | 84 (20.6%) |  |
| Wave2Drinking | N=1920 | N=1512 | N=408 | <0.001 |
| No | 173 (9.0%) | 117 (7.7%) | 56 (13.7%) |  |
| Yes | 1747 (91.0%) | 1395 (92.3%) | 352 (86.3%) | |
| Wave2Self.rated.Health | N=1920 | N=1512 | N=408 | <0.001 |
| Poor | 91 (4.7%) | 29 (1.9%) | 62 (15.2%) |  |
| Fair | 345 (18.0%) | 216 (14.3%) | 129 (31.6%) | |
| Good / Very good | 1484 (77.3%) | 1267 (83.8%) | 217 (53.2%) | |
| Wave2Cancer | N=1920 | N=1512 | N=408 | 0.989 |
| No | 1795 (93.5%) | 1413 (93.5%) | 382 (93.6%) | |
| Yes | 125 (6.5%) | 99 (6.5%) | 26 (6.4%) |  |
| Wave2Arthritis | N=1920 | N=1512 | N=408 | <0.001 |
| No | 1259 (65.6%) | 1059 (70.0%) | 200 (49.0%) | |
| Yes | 661 (34.4%) | 453 (30.0%) | 208 (51.0%) | |
| Wave2Pension | N=1920 | N=1512 | N=408 | 0.001 |
| No | 1352 (70.4%) | 1037 (68.6%) | 315 (77.2%) | |
| Yes | 568 (29.6%) | 475 (31.4%) | 93 (22.8%) |  |
| Wave2Visual.Impairment | N=1920 | N=1512 | N=408 | 0.007 |
| No | 1859 (96.8%) | 1473 (97.4%) | 386 (94.6%) | |
| Yes | 61 (3.2%) | 39 (2.6%) | 22 (5.4%) |  |
| Wave2Hypertension | N=1920 | N=1512 | N=408 | 0.003 |
| No | 1191 (62.0%) | 964 (63.8%) | 227 (55.6%) | |
| Yes | 729 (38.0%) | 548 (36.2%) | 181 (44.4%) | |
| Wave2Diabetes | N=1920 | N=1512 | N=408 | 0.044 |
| No | 1799 (93.7%) | 1426 (94.3%) | 373 (91.4%) | |
| Yes | 121 (6.3%) | 86 (5.7%) | 35 (8.6%) |  |
| Wave2Employment.Status | N=1920 | N=1512 | N=408 | <0.001 |
| Unemployed | 301 (15.7%) | 209 (13.8%) | 92 (22.5%) |  |
| Retired | 990 (51.6%) | 796 (52.6%) | 194 (47.5%) | |
| Working | 629 (32.8%) | 507 (33.5%) | 122 (29.9%) | |
| Wave2SocialActivity | N=1920 | N=1512 | N=408 | <0.001 |
| No | 1099 (57.2%) | 818 (54.1%) | 281 (68.9%) | |
| Yes | 821 (42.8%) | 694 (45.9%) | 127 (31.1%) | |
| Wave2Falls | N=1920 | N=1512 | N=408 | <0.001 |
| No | 1445 (75.3%) | 1179 (78.0%) | 266 (65.2%) | |
| Yes | 475 (24.7%) | 333 (22.0%) | 142 (34.8%) | |
| Wave2ADL | N=1920 | N=1512 | N=408 | <0.001 |
| No | 1567 (81.6%) | 1304 (86.2%) | 263 (64.5%) | |
| Yes | 353 (18.4%) | 208 (13.8%) | 145 (35.5%) | |
| Wave2IADL | N=1920 | N=1512 | N=408 | <0.001 |
| No | 1581 (82.3%) | 1316 (87.0%) | 265 (65.0%) | |
| Yes | 339 (17.7%) | 196 (13.0%) | 143 (35.0%) | |
| Wave2Marital_Status | N=1920 | N=1512 | N=408 | <0.001 |
| No | 596 (31.0%) | 415 (27.4%) | 181 (44.4%) | |
| Yes | 1324 (69.0%) | 1097 (72.6%) | 227 (55.6%) | |
| Wave2Stroke | N=1920 | N=1512 | N=408 | 0.874 |
| No | 1863 (97.0%) | 1467 (97.0%) | 396 (97.1%) | |
| Yes | 57 (3.0%) | 45 (3.0%) | 12 (2.9%) |  |
| Wave2Age | 1.65 ± 0.48 | 1.66 ± 0.48 | 1.62 ± 0.49 | 0.135 |
| Wave2Walking time | 2.88 ± 1.11 | 2.74 ± 0.89 | 3.40 ± 1.59 | <0.001 |
| Wave2SystolicBP | 134.35 ± 17.89 | 134.67 ± 17.63 | 133.13 ± 18.80 | 0.137 |
| Wave2DiastolicBP | 75.64 ± 10.55 | 75.82 ± 10.45 | 74.98 ± 10.88 | 0.162 |
| Wave2LeftGrip | 29.37 ± 10.79 | 30.32 ± 10.66 | 25.82 ± 10.54 | <0.001 |
| Wave2RightGrip | 31.52 ± 11.38 | 32.57 ± 11.05 | 27.64 ± 11.73 | <0.001 |
| Wave2BMI | 28.30 ± 4.90 | 28.01 ± 4.60 | 29.34 ± 5.76 | <0.001 |
| Wave2PeakFlow | 3.23 ± 1.01 | 3.28 ± 1.01 | 3.01 ± 1.01 | <0.001 |
| Wave2FullTandem | 22.26 ± 9.77 | 22.78 ± 9.62 | 20.33 ± 10.09 | <0.001 |

**Supplementary Table 3.** Baseline characteristics of ELSA cohort participants (Wave 4)

| Variable | Overall | Non_Depressed | Depressed | P_Value |
| --- | --- | --- | --- | --- |
| Gender | N=1920 | N=1517 | N=403 | <0.001 |
| Male | 832 (43.3%) | 719 (47.4%) | 113 (28.0%) | |
| Female | 1088 (56.7%) | 798 (52.6%) | 290 (72.0%) | |
| Education | N=1920 | N=1517 | N=403 | <0.001 |
| Less than middle school | 826 (43.0%) | 608 (40.1%) | 218 (54.1%) | |
| High school | 343 (17.9%) | 281 (18.5%) | 62 (15.4%) |  |
| Higher education | 751 (39.1%) | 628 (41.4%) | 123 (30.5%) | |
| Adverse Childhood Experiences | N=1920 | N=1517 | N=403 | <0.001 |
| Yes | 1920 (100.0%) | 1517 (100.0%) | 403 (100.0%) | |
| Wave4HighIntensityPA | N=1920 | N=1517 | N=403 | <0.001 |
| Low frequency | 1330 (69.3%) | 1008 (66.4%) | 322 (79.9%) | |
| High frequency | 590 (30.7%) | 509 (33.6%) | 81 (20.1%) |  |
| Wave4MediumIntensityPA | N=1920 | N=1517 | N=403 | <0.001 |
| Low frequency | 450 (23.4%) | 292 (19.2%) | 158 (39.2%) | |
| High frequency | 1470 (76.6%) | 1225 (80.8%) | 245 (60.8%) | |
| Wave4LowIntensityPA | N=1920 | N=1517 | N=403 | 0.001 |
| Low frequency | 195 (10.2%) | 136 (9.0%) | 59 (14.6%) |  |
| High frequency | 1725 (89.8%) | 1381 (91.0%) | 344 (85.4%) | |
| Wave4Smoking | N=1920 | N=1517 | N=403 | <0.001 |
| Never smoked | 696 (36.2%) | 567 (37.4%) | 129 (32.0%) | |
| Former smoker | 1006 (52.4%) | 811 (53.5%) | 195 (48.4%) | |
| Current smoker | 218 (11.4%) | 139 (9.2%) | 79 (19.6%) |  |
| Wave4Drinking | N=1920 | N=1517 | N=403 | <0.001 |
| No | 232 (12.1%) | 146 (9.6%) | 86 (21.3%) |  |
| Yes | 1688 (87.9%) | 1371 (90.4%) | 317 (78.7%) | |
| Wave4Self.rated.Health | N=1920 | N=1517 | N=403 | <0.001 |
| Poor | 110 (5.7%) | 39 (2.6%) | 71 (17.6%) |  |
| Fair | 395 (20.6%) | 235 (15.5%) | 160 (39.7%) | |
| Good / Very good | 1415 (73.7%) | 1243 (81.9%) | 172 (42.7%) | |
| Wave4Cancer | N=1920 | N=1517 | N=403 | 0.493 |
| No | 1753 (91.3%) | 1389 (91.6%) | 364 (90.3%) | |
| Yes | 167 (8.7%) | 128 (8.4%) | 39 (9.7%) |  |
| Wave4Arthritis | N=1920 | N=1517 | N=403 | <0.001 |
| No | 1120 (58.3%) | 964 (63.5%) | 156 (38.7%) | |
| Yes | 800 (41.7%) | 553 (36.5%) | 247 (61.3%) | |
| Wave4Pension | N=1920 | N=1517 | N=403 | <0.001 |
| No | 1190 (62.0%) | 902 (59.5%) | 288 (71.5%) | |
| Yes | 730 (38.0%) | 615 (40.5%) | 115 (28.5%) | |
| Wave4Visual.Impairment | N=1920 | N=1517 | N=403 | <0.001 |
| No | 1849 (96.3%) | 1474 (97.2%) | 375 (93.1%) | |
| Yes | 71 (3.7%) | 43 (2.8%) | 28 (6.9%) |  |
| Wave4Hypertension | N=1920 | N=1517 | N=403 | 0.008 |
| No | 1043 (54.3%) | 848 (55.9%) | 195 (48.4%) | |
| Yes | 877 (45.7%) | 669 (44.1%) | 208 (51.6%) | |
| Wave4Diabetes | N=1920 | N=1517 | N=403 | 0.024 |
| No | 1740 (90.6%) | 1387 (91.4%) | 353 (87.6%) | |
| Yes | 180 (9.4%) | 130 (8.6%) | 50 (12.4%) |  |
| Wave4Employment.Status | N=1920 | N=1517 | N=403 | <0.001 |
| Unemployed | 216 (11.2%) | 135 (8.9%) | 81 (20.1%) |  |
| Retired | 1253 (65.3%) | 999 (65.9%) | 254 (63.0%) | |
| Working | 451 (23.5%) | 383 (25.2%) | 68 (16.9%) |  |
| Wave4SocialActivity | N=1920 | N=1517 | N=403 | <0.001 |
| No | 1276 (66.5%) | 969 (63.9%) | 307 (76.2%) | |
| Yes | 644 (33.5%) | 548 (36.1%) | 96 (23.8%) |  |
| Wave4Falls | N=1920 | N=1517 | N=403 | <0.001 |
| No | 1447 (75.4%) | 1195 (78.8%) | 252 (62.5%) | |
| Yes | 473 (24.6%) | 322 (21.2%) | 151 (37.5%) | |
| Wave4ADL | N=1920 | N=1517 | N=403 | <0.001 |
| No | 1546 (80.5%) | 1285 (84.7%) | 261 (64.8%) | |
| Yes | 374 (19.5%) | 232 (15.3%) | 142 (35.2%) | |
| Wave4IADL | N=1920 | N=1517 | N=403 | <0.001 |
| No | 1543 (80.4%) | 1297 (85.5%) | 246 (61.0%) | |
| Yes | 377 (19.6%) | 220 (14.5%) | 157 (39.0%) | |
| Wave4Marital_Status | N=1920 | N=1517 | N=403 | <0.001 |
| No | 631 (32.9%) | 433 (28.5%) | 198 (49.1%) | |
| Yes | 1289 (67.1%) | 1084 (71.5%) | 205 (50.9%) | |
| Wave4Stroke | N=1920 | N=1517 | N=403 | 0.984 |
| No | 1838 (95.7%) | 1452 (95.7%) | 386 (95.8%) | |
| Yes | 82 (4.3%) | 65 (4.3%) | 17 (4.2%) |  |
| Wave4Age | 1.85 ± 0.37 | 1.85 ± 0.37 | 1.84 ± 0.39 | 0.552 |
| Wave4Walking time | 3.19 ± 1.56 | 2.99 ± 1.23 | 3.91 ± 2.28 | <0.001 |
| Wave4SystolicBP | 133.75 ± 16.96 | 134.02 ± 16.84 | 132.73 ± 17.37 | 0.184 |
| Wave4DiastolicBP | 73.75 ± 10.65 | 73.92 ± 10.52 | 73.11 ± 11.14 | 0.188 |
| Wave4LeftGrip | 27.49 ± 10.39 | 28.51 ± 10.19 | 23.65 ± 10.25 | <0.001 |
| Wave4RightGrip | 29.58 ± 11.11 | 30.71 ± 10.79 | 25.32 ± 11.25 | <0.001 |
| Wave4BMI | 28.55 ± 5.11 | 28.32 ± 4.86 | 29.39 ± 5.88 | 0.001 |
| Wave4PeakFlow | 3.11 ± 1.03 | 3.21 ± 1.04 | 2.72 ± 0.90 | <0.001 |
| Wave4FullTandem | 19.27 ± 10.35 | 19.67 ± 10.32 | 17.73 ± 10.34 | 0.001 |

**Supplementary Table 4.** Baseline characteristics of ELSA cohort participants (Wave 6)

| Variable | Overall | Non_Depressed | Depressed | P_Value |
| --- | --- | --- | --- | --- |
| Gender | N=1920 | N=1502 | N=418 | <0.001 |
| Male | 832 (43.3%) | 694 (46.2%) | 138 (33.0%) | |
| Female | 1088 (56.7%) | 808 (53.8%) | 280 (67.0%) | |
| Education | N=1920 | N=1502 | N=418 | <0.001 |
| Less than middle school | 826 (43.0%) | 607 (40.4%) | 219 (52.4%) | |
| High school | 343 (17.9%) | 272 (18.1%) | 71 (17.0%) |  |
| Higher education | 751 (39.1%) | 623 (41.5%) | 128 (30.6%) | |
| Adverse Childhood Experiences | N=1920 | N=1502 | N=418 | <0.001 |
| Yes | 1920 (100.0%) | 1502 (100.0%) | 418 (100.0%) | |
| Wave6HighIntensityPA | N=1920 | N=1502 | N=418 | <0.001 |
| Low frequency | 1427 (74.3%) | 1072 (71.4%) | 355 (84.9%) | |
| High frequency | 493 (25.7%) | 430 (28.6%) | 63 (15.1%) |  |
| Wave6MediumIntensityPA | N=1920 | N=1502 | N=418 | <0.001 |
| Low frequency | 560 (29.2%) | 362 (24.1%) | 198 (47.4%) | |
| High frequency | 1360 (70.8%) | 1140 (75.9%) | 220 (52.6%) | |
| Wave6LowIntensityPA | N=1920 | N=1502 | N=418 | <0.001 |
| Low frequency | 253 (13.2%) | 159 (10.6%) | 94 (22.5%) |  |
| High frequency | 1667 (86.8%) | 1343 (89.4%) | 324 (77.5%) | |
| Wave6Smoking | N=1920 | N=1502 | N=418 | <0.001 |
| Never smoked | 623 (32.4%) | 503 (33.5%) | 120 (28.7%) | |
| Former smoker | 1123 (58.5%) | 888 (59.1%) | 235 (56.2%) | |
| Current smoker | 174 (9.1%) | 111 (7.4%) | 63 (15.1%) |  |
| Wave6Drinking | N=1920 | N=1502 | N=418 | <0.001 |
| No | 295 (15.4%) | 205 (13.6%) | 90 (21.5%) |  |
| Yes | 1625 (84.6%) | 1297 (86.4%) | 328 (78.5%) | |
| Wave6Self.rated.Health | N=1920 | N=1502 | N=418 | <0.001 |
| Poor | 170 (8.9%) | 57 (3.8%) | 113 (27.0%) | |
| Fair | 462 (24.1%) | 310 (20.6%) | 152 (36.4%) | |
| Good / Very good | 1288 (67.1%) | 1135 (75.6%) | 153 (36.6%) | |
| Wave6Cancer | N=1920 | N=1502 | N=418 | 0.009 |
| No | 1673 (87.1%) | 1325 (88.2%) | 348 (83.3%) | |
| Yes | 247 (12.9%) | 177 (11.8%) | 70 (16.7%) |  |
| Wave6Arthritis | N=1920 | N=1502 | N=418 | <0.001 |
| No | 1014 (52.8%) | 854 (56.9%) | 160 (38.3%) | |
| Yes | 906 (47.2%) | 648 (43.1%) | 258 (61.7%) | |
| Wave6Pension | N=1920 | N=1502 | N=418 | <0.001 |
| No | 1052 (54.8%) | 782 (52.1%) | 270 (64.6%) | |
| Yes | 868 (45.2%) | 720 (47.9%) | 148 (35.4%) | |
| Wave6Visual.Impairment | N=1920 | N=1502 | N=418 | 0.002 |
| No | 1837 (95.7%) | 1449 (96.5%) | 388 (92.8%) | |
| Yes | 83 (4.3%) | 53 (3.5%) | 30 (7.2%) |  |
| Wave6Hypertension | N=1920 | N=1502 | N=418 | 0.041 |
| No | 937 (48.8%) | 752 (50.1%) | 185 (44.3%) | |
| Yes | 983 (51.2%) | 750 (49.9%) | 233 (55.7%) | |
| Wave6Diabetes | N=1920 | N=1502 | N=418 | 0.001 |
| No | 1689 (88.0%) | 1341 (89.3%) | 348 (83.3%) | |
| Yes | 231 (12.0%) | 161 (10.7%) | 70 (16.7%) |  |
| Wave6Employment.Status | N=1920 | N=1502 | N=418 | <0.001 |
| Unemployed | 152 (7.9%) | 98 (6.5%) | 54 (12.9%) |  |
| Retired | 1523 (79.3%) | 1190 (79.2%) | 333 (79.7%) | |
| Working | 245 (12.8%) | 214 (14.2%) | 31 (7.4%) |  |
| Wave6SocialActivity | N=1920 | N=1502 | N=418 | 0.002 |
| No | 1304 (67.9%) | 993 (66.1%) | 311 (74.4%) | |
| Yes | 616 (32.1%) | 509 (33.9%) | 107 (25.6%) | |
| Wave6Falls | N=1920 | N=1502 | N=418 | <0.001 |
| No | 1347 (70.2%) | 1106 (73.6%) | 241 (57.7%) | |
| Yes | 573 (29.8%) | 396 (26.4%) | 177 (42.3%) | |
| Wave6ADL | N=1920 | N=1502 | N=418 | <0.001 |
| No | 1500 (78.1%) | 1251 (83.3%) | 249 (59.6%) | |
| Yes | 420 (21.9%) | 251 (16.7%) | 169 (40.4%) | |
| Wave6IADL | N=1920 | N=1502 | N=418 | <0.001 |
| No | 1456 (75.8%) | 1234 (82.2%) | 222 (53.1%) | |
| Yes | 464 (24.2%) | 268 (17.8%) | 196 (46.9%) | |
| Wave6Marital_Status | N=1920 | N=1502 | N=418 | <0.001 |
| No | 702 (36.6%) | 490 (32.6%) | 212 (50.7%) | |
| Yes | 1218 (63.4%) | 1012 (67.4%) | 206 (49.3%) | |
| Wave6Stroke | N=1920 | N=1502 | N=418 | 0.093 |
| No | 1804 (94.0%) | 1419 (94.5%) | 385 (92.1%) | |
| Yes | 116 (6.0%) | 83 (5.5%) | 33 (7.9%) |  |
| Wave6Age | 2.01 ± 0.19 | 2.01 ± 0.18 | 2.03 ± 0.22 | 0.018 |
| Wave6Walking time | 3.48 ± 2.15 | 3.20 ± 1.89 | 4.46 ± 2.68 | <0.001 |
| Wave6SystolicBP | 133.63 ± 17.39 | 133.56 ± 17.11 | 133.90 ± 18.40 | 0.733 |
| Wave6DiastolicBP | 71.86 ± 10.52 | 72.13 ± 10.30 | 70.90 ± 11.23 | 0.044 |
| Wave6LeftGrip | 25.71 ± 9.97 | 26.62 ± 9.86 | 22.45 ± 9.68 | <0.001 |
| Wave6RightGrip | 27.56 ± 10.59 | 28.53 ± 10.45 | 24.07 ± 10.37 | <0.001 |
| Wave6BMI | 28.44 ± 4.99 | 28.36 ± 4.89 | 28.72 ± 5.34 | 0.219 |
| Wave6PeakFlow | 3.05 ± 0.92 | 3.13 ± 0.92 | 2.75 ± 0.87 | <0.001 |
| Wave6FullTandem | 16.53 ± 10.18 | 16.98 ± 10.29 | 14.92 ± 9.60 | <0.001 |

**Supplementary Table 5.** Baseline characteristics of HRS cohort participants (Wave 9)

| Variable | Overall | Non_Depressed | Depressed | P_Value |
| --- | --- | --- | --- | --- |
| Gender | N=2015 | N=1575 | N=440 | <0.001 |
| Male | 842 (41.8%) | 702 (44.6%) | 140 (31.8%) | |
| Female | 1173 (58.2%) | 873 (55.4%) | 300 (68.2%) | |
| Education | N=2015 | N=1575 | N=440 | <0.001 |
| Less than middle school | 285 (14.1%) | 174 (11.0%) | 111 (25.2%) | |
| High school | 748 (37.1%) | 582 (37.0%) | 166 (37.7%) | |
| Higher education | 982 (48.7%) | 819 (52.0%) | 163 (37.0%) | |
| Adverse Childhood Experiences | N=2015 | N=1575 | N=440 | <0.001 |
| Yes | 2015 (100.0%) | 1575 (100.0%) | 440 (100.0%) | |
| Wave9HighIntensityPA | N=2015 | N=1575 | N=440 | <0.001 |
| Low frequency | 1236 (61.3%) | 909 (57.7%) | 327 (74.3%) | |
| High frequency | 779 (38.7%) | 666 (42.3%) | 113 (25.7%) | |
| Wave9MediumIntensityPA | N=2015 | N=1575 | N=440 | <0.001 |
| Low frequency | 482 (23.9%) | 329 (20.9%) | 153 (34.8%) | |
| High frequency | 1533 (76.1%) | 1246 (79.1%) | 287 (65.2%) | |
| Wave9LowIntensityPA | N=2015 | N=1575 | N=440 | 0.001 |
| Low frequency | 207 (10.3%) | 142 (9.0%) | 65 (14.8%) |  |
| High frequency | 1808 (89.7%) | 1433 (91.0%) | 375 (85.2%) | |
| Wave9Smoking | N=2015 | N=1575 | N=440 | <0.001 |
| Never smoked | 773 (38.4%) | 610 (38.7%) | 163 (37.0%) | |
| Former smoker | 929 (46.1%) | 761 (48.3%) | 168 (38.2%) | |
| Current smoker | 313 (15.5%) | 204 (13.0%) | 109 (24.8%) | |
| Wave9Drinking | N=2015 | N=1575 | N=440 | <0.001 |
| No | 1135 (56.3%) | 928 (58.9%) | 207 (47.0%) | |
| Yes | 880 (43.7%) | 647 (41.1%) | 233 (53.0%) | |
| Wave9Self.rated.Health | N=2015 | N=1575 | N=440 | <0.001 |
| Poor | 107 (5.3%) | 37 (2.3%) | 70 (15.9%) |  |
| Fair | 370 (18.4%) | 220 (14.0%) | 150 (34.1%) | |
| Good / Very good | 1538 (76.3%) | 1318 (83.7%) | 220 (50.0%) | |
| Wave9Cancer | N=2015 | N=1575 | N=440 | 0.915 |
| No | 1744 (86.6%) | 1362 (86.5%) | 382 (86.8%) | |
| Yes | 271 (13.4%) | 213 (13.5%) | 58 (13.2%) |  |
| Wave9Arthritis | N=2015 | N=1575 | N=440 | <0.001 |
| No | 712 (35.3%) | 594 (37.7%) | 118 (26.8%) | |
| Yes | 1303 (64.7%) | 981 (62.3%) | 322 (73.2%) | |
| Wave9Pension | N=2015 | N=1575 | N=440 | 0.001 |
| No | 1507 (74.8%) | 1151 (73.1%) | 356 (80.9%) | |
| Yes | 508 (25.2%) | 424 (26.9%) | 84 (19.1%) |  |
| Wave9Visual.Impairment | N=2015 | N=1575 | N=440 | <0.001 |
| No | 1609 (79.9%) | 1332 (84.6%) | 277 (63.0%) | |
| Yes | 406 (20.1%) | 243 (15.4%) | 163 (37.0%) | |
| Wave9Hypertension | N=2015 | N=1575 | N=440 | 0.02 |
| No | 971 (48.2%) | 781 (49.6%) | 190 (43.2%) | |
| Yes | 1044 (51.8%) | 794 (50.4%) | 250 (56.8%) | |
| Wave9Diabetes | N=2015 | N=1575 | N=440 | 0.066 |
| No | 1690 (83.9%) | 1334 (84.7%) | 356 (80.9%) | |
| Yes | 325 (16.1%) | 241 (15.3%) | 84 (19.1%) |  |
| Wave9Employment.Status | N=2015 | N=1575 | N=440 | <0.001 |
| Unemployed | 184 (9.1%) | 111 (7.0%) | 73 (16.6%) |  |
| Retired | 842 (41.8%) | 647 (41.1%) | 195 (44.3%) | |
| Working | 989 (49.1%) | 817 (51.9%) | 172 (39.1%) | |
| Wave9SocialActivity | N=2015 | N=1575 | N=440 | 0.018 |
| No | 388 (19.3%) | 321 (20.4%) | 67 (15.2%) |  |
| Yes | 1627 (80.7%) | 1254 (79.6%) | 373 (84.8%) | |
| Wave9Falls | N=2015 | N=1575 | N=440 | <0.001 |
| No | 1589 (78.9%) | 1280 (81.3%) | 309 (70.2%) | |
| Yes | 426 (21.1%) | 295 (18.7%) | 131 (29.8%) | |
| Wave9ADL | N=2015 | N=1575 | N=440 | <0.001 |
| No | 1786 (88.6%) | 1471 (93.4%) | 315 (71.6%) | |
| Yes | 229 (11.4%) | 104 (6.6%) | 125 (28.4%) | |
| Wave9IADL | N=2015 | N=1575 | N=440 | <0.001 |
| No | 1855 (92.1%) | 1514 (96.1%) | 341 (77.5%) | |
| Yes | 160 (7.9%) | 61 (3.9%) | 99 (22.5%) |  |
| Wave9Marital_Status | N=2015 | N=1575 | N=440 | <0.001 |
| No | 712 (35.3%) | 502 (31.9%) | 210 (47.7%) | |
| Yes | 1303 (64.7%) | 1073 (68.1%) | 230 (52.3%) | |
| Wave9Stroke | N=2015 | N=1575 | N=440 | <0.001 |
| No | 1908 (94.7%) | 1510 (95.9%) | 398 (90.5%) | |
| Yes | 107 (5.3%) | 65 (4.1%) | 42 (9.5%) |  |
| Wave9Sleep | N=2015 | N=1575 | N=440 | <0.001 |
| No | 1506 (74.7%) | 1239 (78.7%) | 267 (60.7%) | |
| Yes | 509 (25.3%) | 336 (21.3%) | 173 (39.3%) | |
| Wave9Age | 1.67 ± 0.47 | 1.68 ± 0.47 | 1.61 ± 0.49 | 0.006 |
| Wave9Walking time | 3.28 ± 0.88 | 3.16 ± 0.77 | 3.72 ± 1.07 | <0.001 |
| Wave9SystolicBP | 127.01 ± 14.46 | 127.43 ± 14.55 | 125.49 ± 14.05 | 0.011 |
| Wave9DiastolicBP | 79.30 ± 7.84 | 79.55 ± 7.68 | 78.39 ± 8.32 | 0.008 |
| Wave9LeftGrip | 28.70 ± 8.13 | 29.36 ± 8.10 | 26.34 ± 7.82 | <0.001 |
| Wave9RightGrip | 31.39 ± 8.45 | 32.08 ± 8.45 | 28.89 ± 7.97 | <0.001 |
| Wave9BMI | 30.26 ± 4.49 | 30.02 ± 4.36 | 31.12 ± 4.80 | <0.001 |
| Wave9PeakFlow | 370.21 ± 94.57 | 378.62 ± 94.44 | 340.10 ± 88.82 | <0.001 |
| Wave9FullTandem | 47.36 ± 15.67 | 48.67 ± 14.99 | 42.67 ± 17.10 | <0.001 |

**Supplementary Table 6.** Baseline characteristics of HRS cohort participants (Wave 11)

| Variable | Overall | Non_Depressed | Depressed | P_Value |
| --- | --- | --- | --- | --- |
| Gender | N=2015 | N=1583 | N=432 | <0.001 |
| Male | 842 (41.8%) | 699 (44.2%) | 143 (33.1%) | |
| Female | 1173 (58.2%) | 884 (55.8%) | 289 (66.9%) | |
| Education | N=2015 | N=1583 | N=432 | <0.001 |
| Less than middle school | 285 (14.1%) | 171 (10.8%) | 114 (26.4%) | |
| High school | 748 (37.1%) | 592 (37.4%) | 156 (36.1%) | |
| Higher education | 982 (48.7%) | 820 (51.8%) | 162 (37.5%) | |
| Adverse Childhood Experiences | N=2015 | N=1583 | N=432 | <0.001 |
| Yes | 2015 (100.0%) | 1583 (100.0%) | 432 (100.0%) | |
| Wave11HighIntensityPA | N=2015 | N=1583 | N=432 | <0.001 |
| Low frequency | 1231 (61.1%) | 903 (57.0%) | 328 (75.9%) | |
| High frequency | 784 (38.9%) | 680 (43.0%) | 104 (24.1%) | |
| Wave11MediumIntensityPA | N=2015 | N=1583 | N=432 | <0.001 |
| Low frequency | 612 (30.4%) | 419 (26.5%) | 193 (44.7%) | |
| High frequency | 1403 (69.6%) | 1164 (73.5%) | 239 (55.3%) | |
| Wave11LowIntensityPA | N=2015 | N=1583 | N=432 | <0.001 |
| Low frequency | 287 (14.2%) | 184 (11.6%) | 103 (23.8%) | |
| High frequency | 1728 (85.8%) | 1399 (88.4%) | 329 (76.2%) | |
| Wave11Smoking | N=2015 | N=1583 | N=432 | <0.001 |
| Never smoked | 773 (38.4%) | 625 (39.5%) | 148 (34.3%) | |
| Former smoker | 984 (48.8%) | 796 (50.3%) | 188 (43.5%) | |
| Current smoker | 258 (12.8%) | 162 (10.2%) | 96 (22.2%) |  |
| Wave11Drinking | N=2015 | N=1583 | N=432 | <0.001 |
| No | 1131 (56.1%) | 931 (58.8%) | 200 (46.3%) | |
| Yes | 884 (43.9%) | 652 (41.2%) | 232 (53.7%) | |
| Wave11Self.rated.Health | N=2015 | N=1583 | N=432 | <0.001 |
| Poor | 108 (5.4%) | 39 (2.5%) | 69 (16.0%) |  |
| Fair | 369 (18.3%) | 209 (13.2%) | 160 (37.0%) | |
| Good / Very good | 1538 (76.3%) | 1335 (84.3%) | 203 (47.0%) | |
| Wave11Cancer | N=2015 | N=1583 | N=432 | 0.285 |
| No | 1665 (82.6%) | 1316 (83.1%) | 349 (80.8%) | |
| Yes | 350 (17.4%) | 267 (16.9%) | 83 (19.2%) |  |
| Wave11Arthritis | N=2015 | N=1583 | N=432 | <0.001 |
| No | 555 (27.5%) | 472 (29.8%) | 83 (19.2%) |  |
| Yes | 1460 (72.5%) | 1111 (70.2%) | 349 (80.8%) | |
| Wave11Pension | N=2015 | N=1583 | N=432 | 0.001 |
| No | 1391 (69.0%) | 1064 (67.2%) | 327 (75.7%) | |
| Yes | 624 (31.0%) | 519 (32.8%) | 105 (24.3%) | |
| Wave11Visual.Impairment | N=2015 | N=1583 | N=432 | <0.001 |
| No | 1568 (77.8%) | 1299 (82.1%) | 269 (62.3%) | |
| Yes | 447 (22.2%) | 284 (17.9%) | 163 (37.7%) | |
| Wave11Hypertension | N=2015 | N=1583 | N=432 | <0.001 |
| No | 826 (41.0%) | 687 (43.4%) | 139 (32.2%) | |
| Yes | 1189 (59.0%) | 896 (56.6%) | 293 (67.8%) | |
| Wave11Diabetes | N=2015 | N=1583 | N=432 | 0.066 |
| No | 1561 (77.5%) | 1241 (78.4%) | 320 (74.1%) | |
| Yes | 454 (22.5%) | 342 (21.6%) | 112 (25.9%) | |
| Wave11Employment.Status | N=2015 | N=1583 | N=432 | <0.001 |
| Unemployed | 122 (6.1%) | 81 (5.1%) | 41 (9.5%) |  |
| Retired | 1156 (57.4%) | 881 (55.7%) | 275 (63.7%) | |
| Working | 737 (36.6%) | 621 (39.2%) | 116 (26.9%) | |
| Wave11SocialActivity | N=2015 | N=1583 | N=432 | <0.001 |
| No | 1029 (51.1%) | 760 (48.0%) | 269 (62.3%) | |
| Yes | 986 (48.9%) | 823 (52.0%) | 163 (37.7%) | |
| Wave11Falls | N=2015 | N=1583 | N=432 | <0.001 |
| No | 1501 (74.5%) | 1214 (76.7%) | 287 (66.4%) | |
| Yes | 514 (25.5%) | 369 (23.3%) | 145 (33.6%) | |
| Wave11ADL | N=2015 | N=1583 | N=432 | <0.001 |
| No | 1736 (86.2%) | 1442 (91.1%) | 294 (68.1%) | |
| Yes | 279 (13.8%) | 141 (8.9%) | 138 (31.9%) | |
| Wave11IADL | N=2015 | N=1583 | N=432 | <0.001 |
| No | 1802 (89.4%) | 1482 (93.6%) | 320 (74.1%) | |
| Yes | 213 (10.6%) | 101 (6.4%) | 112 (25.9%) | |
| Wave11Marital_Status | N=2015 | N=1583 | N=432 | <0.001 |
| No | 796 (39.5%) | 547 (34.6%) | 249 (57.6%) | |
| Yes | 1219 (60.5%) | 1036 (65.4%) | 183 (42.4%) | |
| Wave11Stroke | N=2015 | N=1583 | N=432 | <0.001 |
| No | 1859 (92.3%) | 1481 (93.6%) | 378 (87.5%) | |
| Yes | 156 (7.7%) | 102 (6.4%) | 54 (12.5%) |  |
| Wave11Sleep | N=2015 | N=1583 | N=432 | <0.001 |
| No | 1473 (73.1%) | 1251 (79.0%) | 222 (51.4%) | |
| Yes | 542 (26.9%) | 332 (21.0%) | 210 (48.6%) | |
| Wave11Age | 1.88 ± 0.34 | 1.88 ± 0.34 | 1.87 ± 0.36 | 0.712 |
| Wave11Walking time | 3.22 ± 0.76 | 3.09 ± 0.69 | 3.69 ± 0.85 | <0.001 |
| Wave11SystolicBP | 126.90 ± 14.24 | 126.74 ± 14.22 | 127.50 ± 14.30 | 0.328 |
| Wave11DiastolicBP | 77.29 ± 7.91 | 77.29 ± 7.78 | 77.27 ± 8.37 | 0.97 |
| Wave11LeftGrip | 26.51 ± 7.69 | 27.25 ± 7.68 | 23.82 ± 7.15 | <0.001 |
| Wave11RightGrip | 29.30 ± 7.99 | 30.11 ± 7.94 | 26.31 ± 7.49 | <0.001 |
| Wave11BMI | 30.26 ± 4.56 | 29.99 ± 4.43 | 31.28 ± 4.88 | <0.001 |
| Wave11PeakFlow | 360.24 ± 96.45 | 371.08 ± 94.89 | 320.52 ± 91.69 | <0.001 |
| Wave11FullTandem | 40.53 ± 16.84 | 41.69 ± 16.59 | 36.26 ± 17.06 | <0.001 |

**Supplementary Table 7.** Baseline characteristics of HRS cohort participants (Wave 13)

| Variable | Overall | Non_Depressed | Depressed | P_Value |
| --- | --- | --- | --- | --- |
| Gender | N=2015 | N=1554 | N=461 | <0.001 |
| Male | 842 (41.8%) | 684 (44.0%) | 158 (34.3%) | |
| Female | 1173 (58.2%) | 870 (56.0%) | 303 (65.7%) | |
| Education | N=2015 | N=1554 | N=461 | <0.001 |
| Less than middle school | 285 (14.1%) | 179 (11.5%) | 106 (23.0%) | |
| High school | 748 (37.1%) | 582 (37.5%) | 166 (36.0%) | |
| Higher education | 982 (48.7%) | 793 (51.0%) | 189 (41.0%) | |
| Adverse Childhood Experiences | N=2015 | N=1554 | N=461 | <0.001 |
| Yes | 2015 (100.0%) | 1554 (100.0%) | 461 (100.0%) | |
| Wave13HighIntensityPA | N=2015 | N=1554 | N=461 | <0.001 |
| Low frequency | 1361 (67.5%) | 984 (63.3%) | 377 (81.8%) | |
| High frequency | 654 (32.5%) | 570 (36.7%) | 84 (18.2%) |  |
| Wave13MediumIntensityPA | N=2015 | N=1554 | N=461 | <0.001 |
| Low frequency | 693 (34.4%) | 462 (29.7%) | 231 (50.1%) | |
| High frequency | 1322 (65.6%) | 1092 (70.3%) | 230 (49.9%) | |
| Wave13LowIntensityPA | N=2015 | N=1554 | N=461 | <0.001 |
| Low frequency | 397 (19.7%) | 247 (15.9%) | 150 (32.5%) | |
| High frequency | 1618 (80.3%) | 1307 (84.1%) | 311 (67.5%) | |
| Wave13Smoking | N=2015 | N=1554 | N=461 | <0.001 |
| Never smoked | 773 (38.4%) | 610 (39.3%) | 163 (35.4%) | |
| Former smoker | 1042 (51.7%) | 820 (52.8%) | 222 (48.2%) | |
| Current smoker | 200 (9.9%) | 124 (8.0%) | 76 (16.5%) |  |
| Wave13Drinking | N=2015 | N=1554 | N=461 | <0.001 |
| No | 1080 (53.6%) | 891 (57.3%) | 189 (41.0%) | |
| Yes | 935 (46.4%) | 663 (42.7%) | 272 (59.0%) | |
| Wave13Self.rated.Health | N=2015 | N=1554 | N=461 | <0.001 |
| Poor | 148 (7.3%) | 55 (3.5%) | 93 (20.2%) |  |
| Fair | 422 (20.9%) | 253 (16.3%) | 169 (36.7%) | |
| Good / Very good | 1445 (71.7%) | 1246 (80.2%) | 199 (43.2%) | |
| Wave13Cancer | N=2015 | N=1554 | N=461 | 0.192 |
| No | 1589 (78.9%) | 1236 (79.5%) | 353 (76.6%) | |
| Yes | 426 (21.1%) | 318 (20.5%) | 108 (23.4%) | |
| Wave13Arthritis | N=2015 | N=1554 | N=461 | <0.001 |
| No | 443 (22.0%) | 380 (24.5%) | 63 (13.7%) |  |
| Yes | 1572 (78.0%) | 1174 (75.5%) | 398 (86.3%) | |
| Wave13Pension | N=2015 | N=1554 | N=461 | 0.007 |
| No | 1271 (63.1%) | 955 (61.5%) | 316 (68.5%) | |
| Yes | 744 (36.9%) | 599 (38.5%) | 145 (31.5%) | |
| Wave13Visual.Impairment | N=2015 | N=1554 | N=461 | <0.001 |
| No | 1504 (74.6%) | 1242 (79.9%) | 262 (56.8%) | |
| Yes | 511 (25.4%) | 312 (20.1%) | 199 (43.2%) | |
| Wave13Hypertension | N=2015 | N=1554 | N=461 | 0.001 |
| No | 702 (34.8%) | 571 (36.7%) | 131 (28.4%) | |
| Yes | 1313 (65.2%) | 983 (63.3%) | 330 (71.6%) | |
| Wave13Diabetes | N=2015 | N=1554 | N=461 | 0.109 |
| No | 1443 (71.6%) | 1127 (72.5%) | 316 (68.5%) | |
| Yes | 572 (28.4%) | 427 (27.5%) | 145 (31.5%) | |
| Wave13Employment.Status | N=2015 | N=1554 | N=461 | <0.001 |
| Unemployed | 87 (4.3%) | 49 (3.2%) | 38 (8.2%) |  |
| Retired | 1440 (71.5%) | 1090 (70.1%) | 350 (75.9%) | |
| Working | 488 (24.2%) | 415 (26.7%) | 73 (15.8%) |  |
| Wave13SocialActivity | N=2015 | N=1554 | N=461 | <0.001 |
| No | 1039 (51.6%) | 724 (46.6%) | 315 (68.3%) | |
| Yes | 976 (48.4%) | 830 (53.4%) | 146 (31.7%) | |
| Wave13Falls | N=2015 | N=1554 | N=461 | <0.001 |
| No | 1321 (65.6%) | 1082 (69.6%) | 239 (51.8%) | |
| Yes | 694 (34.4%) | 472 (30.4%) | 222 (48.2%) | |
| Wave13ADL | N=2015 | N=1554 | N=461 | <0.001 |
| No | 1649 (81.8%) | 1359 (87.5%) | 290 (62.9%) | |
| Yes | 366 (18.2%) | 195 (12.5%) | 171 (37.1%) | |
| Wave13IADL | N=2015 | N=1554 | N=461 | <0.001 |
| No | 1702 (84.5%) | 1404 (90.3%) | 298 (64.6%) | |
| Yes | 313 (15.5%) | 150 (9.7%) | 163 (35.4%) | |
| Wave13Marital_Status | N=2015 | N=1554 | N=461 | <0.001 |
| No | 894 (44.4%) | 616 (39.6%) | 278 (60.3%) | |
| Yes | 1121 (55.6%) | 938 (60.4%) | 183 (39.7%) | |
| Wave13Stroke | N=2015 | N=1554 | N=461 | <0.001 |
| No | 1796 (89.1%) | 1412 (90.9%) | 384 (83.3%) | |
| Yes | 219 (10.9%) | 142 (9.1%) | 77 (16.7%) |  |
| Wave13Sleep | N=2015 | N=1554 | N=461 | <0.001 |
| No | 1698 (84.3%) | 1378 (88.7%) | 320 (69.4%) | |
| Yes | 317 (15.7%) | 176 (11.3%) | 141 (30.6%) | |
| Wave13Age | 2.00 ± 0.20 | 2.00 ± 0.18 | 1.99 ± 0.27 | 0.472 |
| Wave13Walking time | 3.75 ± 1.41 | 3.52 ± 1.14 | 4.53 ± 1.88 | <0.001 |
| Wave13SystolicBP | 126.77 ± 13.94 | 126.39 ± 13.67 | 128.08 ± 14.75 | 0.028 |
| Wave13DiastolicBP | 75.32 ± 7.48 | 75.33 ± 7.11 | 75.31 ± 8.61 | 0.958 |
| Wave13LeftGrip | 24.25 ± 7.06 | 24.96 ± 7.09 | 21.85 ± 6.40 | <0.001 |
| Wave13RightGrip | 26.67 ± 7.53 | 27.39 ± 7.51 | 24.22 ± 7.08 | <0.001 |
| Wave13BMI | 30.13 ± 4.55 | 29.92 ± 4.38 | 30.83 ± 5.01 | <0.001 |
| Wave13PeakFlow | 329.51 ± 100.74 | 342.18 ± 99.56 | 286.80 ± 92.71 | <0.001 |
| Wave13FullTandem | 33.91 ± 16.35 | 35.05 ± 16.30 | 30.05 ± 15.94 | <0.001 |

**Supplementary Table 8.** Stability of baseline predictor selection across 20 multiply imputed datasets in the ELSA development cohort

| Predictor | Selection frequency. n/20 | selected |
| --- | --- | --- |
| Falls | 20/20 (100%) | YES |
| Walking time | 20/20 (100%) | YES |
| Right-hand grip strength | 20/20 (100%) | YES |
| Arthritis | 20/20 (100%) | YES |
| Employment status | 17/20 (85%) | YES |
| Smoking status | 16/20 (80%) | YES |
| HighIntensityPA | 16/20 (80%) | YES |
| MediumIntensityPA | 15/20 (75%) | YES |
| Marital status | 15/20 (75%) | YES |
| Diabetes | 11/20 (55%) | YES |
| Hypertension | 10/20 (50%) | YES |
| Full tandem stand | 9/20 (45%) | NO |
| Peak expiratory flow | 9/20 (45%) | NO |
| BMI | 9/20 (45%) | NO |
| Diastolic blood pressure | 8/20 (40%) | NO |
| Systolic blood pressure | 8/20 (40%) | NO |
| Left-hand grip strength | 5/20 (25%) | NO |
| LowIntensityPA | 5/20 (25%) | NO |
| Visual impairment | 5/20 (25%) | NO |
| Pension | 4/20 (20%) | NO |
| Drinking | 4/20 (20%) | NO |
| Cancer | 4/20 (20%) | NO |
| Stroke | 4/20 (20%) | NO |
| Age | 4/20 (20%) | NO |
| Gender | 3/20 (15%) | NO |
| Education | 3/20 (15%) | NO |

**Supplementary Table 9.** Comprehensive Predictive Performance of 12 Machine Learning Models in ELSA Internal Validation and HRS External Validation

| Model | Dataset | AUC(95% CI) | Acc(95% CI) | Prec(95% CI) | Rec(95% CI) | F1(95% CI) |
| --- | --- | --- | --- | --- | --- | --- |
| Random Forest | ELSA | 0.7011 (0.6720-0.7272) | 0.7818 (0.7625-0.8010) | 0.4933 (0.3810-0.6029) | 0.0885 (0.0634-0.1166) | 0.1501 (0.1086-0.1941) |
|  | HRS | 0.6934 (0.6659-0.7203) | 0.7777 (0.7608-0.7960) | 0.6140 (0.4799-0.7358) | 0.0759 (0.0526-0.1007) | 0.1351 (0.0956-0.1752) |
| AdaBoost | ELSA | 0.6979 (0.6687-0.7242) | 0.7844 (0.7661-0.8026) | 0.5222 (0.4176-0.6186) | 0.1124 (0.0831-0.1439) | 0.1850 (0.1398-0.2310) |
|  | HRS | 0.7049 (0.6812-0.7315) | 0.7816 (0.7643-0.7985) | 0.6364 (0.5285-0.7403) | 0.1063 (0.0788-0.1343) | 0.1822 (0.1376-0.2251) |
| LDA | ELSA | 0.6967 (0.6684-0.7248) | 0.7891 (0.7703-0.8078) | 0.5537 (0.4615-0.6415) | 0.1603 (0.1281-0.1959) | 0.2486 (0.2016-0.2953) |
|  | HRS | 0.6726 (0.6433-0.7022) | 0.7806 (0.7638-0.7980) | 0.5704 (0.4836-0.6541) | 0.1670 (0.1300-0.2010) | 0.2584 (0.2076-0.3038) |
| Logistic Regression | ELSA | 0.6960 (0.6686-0.7238) | 0.7849 (0.7651-0.8026) | 0.5287 (0.4216-0.6283) | 0.1100 (0.0810-0.1411) | 0.1822 (0.1375-0.2267) |
|  | HRS | 0.6686 (0.6386-0.6985) | 0.7836 (0.7663-0.8010) | 0.6263 (0.5288-0.7248) | 0.1345 (0.1032-0.1649) | 0.2214 (0.1747-0.2662) |
| Naive Bayes | ELSA | 0.6909 (0.6631-0.7196) | 0.7406 (0.7224-0.7604) | 0.3925 (0.3411-0.4471) | 0.3493 (0.3046-0.3972) | 0.3696 (0.3248-0.4134) |
|  | HRS | 0.6534 (0.6254-0.6823) | 0.6913 (0.6719-0.7122) | 0.3555 (0.3152-0.3970) | 0.4295 (0.3824-0.4775) | 0.3890 (0.3516-0.4280) |
| QDA | ELSA | 0.6791 (0.6486-0.7076) | 0.7583 (0.7401-0.7776) | 0.4154 (0.3546-0.4800) | 0.2703 (0.2302-0.3142) | 0.3275 (0.2819-0.3731) |
|  | HRS | 0.6449 (0.6167-0.6734) | 0.7042 (0.6839-0.7256) | 0.3490 (0.3045-0.3938) | 0.3384 (0.2937-0.3823) | 0.3436 (0.3010-0.3835) |
| XGBoost | ELSA | 0.6728 (0.6427-0.7005) | 0.7792 (0.7609-0.7990) | 0.4773 (0.3874-0.5630) | 0.1507 (0.1152-0.1871) | 0.2291 (0.1788-0.2767) |
|  | HRS | 0.6711 (0.6428-0.6977) | 0.7797 (0.7618-0.7975) | 0.5739 (0.4790-0.6609) | 0.1432 (0.1113-0.1735) | 0.2292 (0.1818-0.2720) |
| Gradient Boosting | ELSA | 0.6553 (0.6248-0.6832) | 0.7615 (0.7422-0.7813) | 0.3810 (0.3066-0.4540) | 0.1531 (0.1187-0.1888) | 0.2184 (0.1730-0.2612) |
|  | HRS | 0.6446 (0.6152-0.6722) | 0.7737 (0.7558-0.7916) | 0.5159 (0.4359-0.5915) | 0.1757 (0.1413-0.2101) | 0.2621 (0.2164-0.3060) |
| LightGBM | ELSA | 0.6418 (0.6125-0.6684) | 0.7474 (0.7276-0.7667) | 0.3470 (0.2827-0.4126) | 0.1818 (0.1450-0.2215) | 0.2386 (0.1954-0.2828) |
|  | HRS | 0.6271 (0.5977-0.6575) | 0.7385 (0.7191-0.7563) | 0.3680 (0.3093-0.4274) | 0.1996 (0.1635-0.2384) | 0.2588 (0.2142-0.3005) |
| Decision Tree | ELSA | 0.6417 (0.6102-0.6713) | 0.7620 (0.7438-0.7818) | 0.3465 (0.2672-0.4338) | 0.1053 (0.0781-0.1354) | 0.1615 (0.1202-0.2037) |
|  | HRS | 0.6542 (0.6274-0.6822) | 0.7558 (0.7365-0.7752) | 0.3798 (0.2946-0.4616) | 0.1063 (0.0786-0.1360) | 0.1661 (0.1250-0.2073) |
| KNN | ELSA | 0.6391 (0.6080-0.6672) | 0.7693 (0.7495-0.7870) | 0.4339 (0.3602-0.5029) | 0.1962 (0.1575-0.2329) | 0.2702 (0.2215-0.3154) |
|  | HRS | 0.6349 (0.6046-0.6641) | 0.7593 (0.7395-0.7772) | 0.4483 (0.3840-0.5127) | 0.2256 (0.1881-0.2644) | 0.3001 (0.2566-0.3446) |
| SVM | ELSA | 0.6112 (0.5768-0.6446) | 0.7807 (0.7614-0.7995) | 0.4746 (0.3454-0.5938) | 0.0670 (0.0439-0.0911) | 0.1174 (0.0786-0.1560) |
|  | HRS | 0.6385 (0.6068-0.6701) | 0.7782 (0.7613-0.7960) | 0.6522 (0.5000-0.7873) | 0.0651 (0.0436-0.0873) | 0.1183 (0.0811-0.1565) |

**Supplementary Table 10.** Unadjusted and adjusted associations between activity-function transition profiles and incident depression

| Cohort | Model | term | OR | OR_lo | OR_hi | p |
| --- | --- | --- | --- | --- | --- | --- |
| ELSA | Unadjusted | Improving | 1.872087 | 1.077734 | 3.251924 | 0.026036 |
| ELSA | Unadjusted | Declining | 1.671934 | 1.020423 | 2.739418 | 0.041329 |
| ELSA | Unadjusted | Persistently unfavorable | 3.215604 | 2.213722 | 4.670913 | <0.001 |
| ELSA | Adjusted | Improving | 1.6785 | 0.955938 | 2.947223 | 0.071376 |
| ELSA | Adjusted | Declining | 1.484453 | 0.893366 | 2.466624 | 0.127321 |
| ELSA | Adjusted | Persistently unfavorable | 2.591741 | 1.691076 | 3.972101 | <0.001 |
| HRS | Unadjusted | Improving | 0.89416 | 0.421832 | 1.895356 | 0.770399 |
| HRS | Unadjusted | Declining | 2.243514 | 1.448325 | 3.475293 | 0.000296 |
| HRS | Unadjusted | Persistently unfavorable | 2.488343 | 1.71132 | 3.618171 | <0.001 |
| HRS | Adjusted | Improving | 0.84105 | 0.394134 | 1.794735 | 0.654429 |
| HRS | Adjusted | Declining | 2.185687 | 1.3882 | 3.441312 | 0.000735 |
| HRS | Adjusted | Persistently unfavorable | 2.335643 | 1.536503 | 3.550419 | <0.001 |

**Supplementary Table 11.** Sensitivity analysis comparing the primary incident-depression sample with the full analytical sample

| Cohort | Profile | Primary (baseline-depression-free) OR (95%CI) | Primary p | Sensitivity (full sample) OR (95%CI) | Sensitivity p |
| --- | --- | --- | --- | --- | --- |
| ELSA | Improving | 1.68 (0.96–2.95) | 0.071 | 2.16 (1.39–3.37) | 0.001 |
| ELSA | Declining | 1.48 (0.89–2.47) | 0.127 | 1.74 (1.17–2.59) | 0.006 |
| ELSA | Persistently unfavorable | 2.59 (1.69–3.97) | 0 | 3.60 (2.60–4.99) | 0 |
| HRS | Improving | 0.84 (0.39–1.79) | 0.654 | 1.17 (0.68–2.03) | 0.574 |
| HRS | Declining | 2.19 (1.39–3.44) | 0.001 | 1.91 (1.34–2.72) | 0 |
| HRS | Persistently unfavorable | 2.34 (1.54–3.55) | 0 | 3.40 (2.49–4.65) | 0 |

**Supplementary Table 12.** Interaction tests between ACE burden and activity-function transition profiles for incident depression in ELSA and HRS

| Cohort | ACE-burden classification | Likelihood-ratio χ² | df | P for interaction |
| --- | --- | --- | --- | --- |
| ELSA | 1 vs. ≥2 ACEs | 0.113 | 3 | 0.990 |
| ELSA | 1, 2, and ≥3 ACEs | 3.531 | 6 | 0.740 |
| HRS | 1 vs. ≥2 ACEs | 0.877 | 3 | 0.831 |
| HRS | 1, 2, and ≥3 ACEs | 6.901 | 6 | 0.330 |

**Supplementary Table 13.** Baseline characteristics according to predictor-data completeness in ELSA

| Variable | Complete predictor data (n=277) | ≥1 missing predictor value (n=1,643) | Abs. SMD |
| --- | --- | --- | --- |
| Age group (1/2/3), % | 0.0 / 100.0 / 0.0 | 41.1 / 58.8 / 0.1 | 1.184 |
| Female, % | 48.0 | 58.1 | 0.204 |
| Education (1/2/3), % | 30.3 / 23.8 / 45.8 | 42.5 / 17.8 / 39.7 | 0.260 |
| Marital status (partnered), % | 74.0 | 68.1 | 0.130 |
| Employment status (1/2/3), % | 8.7 / 73.6 / 17.7 | 16.8 / 47.8 / 35.3 | 0.548 |
| Smoking (1/2/3), % | 39.7 / 51.6 / 8.7 | 35.7 / 49.8 / 14.5 | 0.187 |
| Walking time, mean (SD) | 2.58 (0.93) | 3.07 (1.35) | 0.386 |
| Left grip strength, mean (SD) | 30.77 (9.98) | 29.33 (11.15) | 0.131 |
| Right-hand grip strength, mean (SD) | 32.73 (10.39) | 31.53 (11.78) | 0.103 |
| Falls, % | 29.6 | 34.7 | 0.109 |
| Arthritis, % | 27.4 | 35.6 | 0.176 |
| Diabetes, % | 5.1 | 6.5 | 0.062 |
| Hypertension, % | 35.7 | 38.3 | 0.054 |
| Moderate-intensity PA, % | 91.0 | 77.8 | 0.363 |
| Vigorous-intensity PA, % | 39.4 | 32.4 | 0.144 |

**Supplementary Table 14.** Baseline characteristics according to predictor-data completeness in HRS

| Variable | Complete cases (n=165) | ≥1 missing predictor (n=1,850) | Abs. SMD |
| --- | --- | --- | --- |
| Age group (1/2), % | 0.0 / 100.0 | 36.5 / 63.5 | 1.072 |
| Female, % | 43.0 | 59.6 | 0.335 |
| Education (1/2/3), % | 10.9 / 44.2 / 44.8 | 14.4 / 36.5 / 49.1 | 0.170 |
| Marital status (partnered), % | 73.3 | 63.9 | 0.204 |
| Employment status (1/2/3), % | 4.8 / 59.4 / 35.8 | 9.5 / 40.2 / 50.3 | 0.399 |
| Smoking (1/2/3), % | 38.8 / 55.8 / 5.5 | 37.9 / 45.5 / 16.6 | 0.371 |
| Walking time, mean (SD) | 3.12 (1.34) | 3.54 (1.51) | 0.291 |
| Left grip strength, mean (SD) | 31.35 (10.08) | 29.92 (11.12) | 0.131 |
| Right-hand grip strength, mean (SD) | 34.51 (10.92) | 32.48 (11.64) | 0.176 |
| Falls, % | 33.9 | 36.0 | 0.042 |
| Arthritis, % | 67.9 | 64.4 | 0.074 |
| Diabetes, % | 15.8 | 16.2 | 0.011 |
| Hypertension, % | 53.3 | 51.7 | 0.033 |
| Moderate-intensity PA, % | 83.0 | 75.4 | 0.187 |
| Vigorous-intensity PA, % | 50.3 | 37.6 | 0.256 |

**Supplementary Table 15.** Association between cumulative ACE burden and incident depressive symptoms in ELSA and HRS

| Cohort | ACE burden | N | Incident depressive symptoms, n (%) | OR (95% CI) | P value |
| --- | --- | --- | --- | --- | --- |
| ELSA | 1 ACE (ref) | 1248 | 254 (20.4) | 1.00 (Reference) | — |
| ELSA | 2 ACEs | 496 | 117 (23.6) | 1.23 (0.96–1.58) | 0.109 |
| ELSA | ≥3 ACEs | 176 | 47 (26.7) | 1.44 (1.00–2.08) | 0.051 |
| ELSA | P for trend* | — | — | 1.21 (1.03–1.42)* | 0.022 |
| HRS | 1 ACE (ref) | 1572 | 351 (22.3) | 1.00 (Reference) | — |
| HRS | 2 ACEs | 401 | 97 (24.2) | 1.09 (0.84–1.42) | 0.518 |
| HRS | ≥3 ACEs | 42 | 13 (31.0) | 1.60 (0.81–3.17) | 0.180 |
| HRS | P for trend* | — | — | 1.15 (0.92–1.43)* | 0.216 |
